# Recognition of candidate transcription factors related to bilberry fruit ripening by *de novo* transcriptome and qRT-PCR analyses

**DOI:** 10.1038/s41598-018-28158-7

**Published:** 2018-07-02

**Authors:** Nga Nguyen, Marko Suokas, Katja Karppinen, Jaana Vuosku, Laura Jaakola, Hely Häggman

**Affiliations:** 1 Department of Ecology and Genetics, University of Oulu, FI-90014 Oulu, Finland; 2 Biocenter Oulu, University of Oulu, FI-90014 Oulu, Finland; 3 Climate laboratory Holt, Department of Arctic and Marine Biology, UiT the Arctic University of Norway, NO-9037 Tromsø, Norway; 4 NIBIO, Norwegian Institute of Bioeconomy Research, NO-1431 Ås, Norway

## Abstract

Bilberry (*Vaccinium myrtillus* L.) fruits are an excellent natural resource for human diet because of their special flavor, taste and nutritional value as well as medical properties. Bilberries are recognized for their high anthocyanin content and many of the genes involved in the anthocyanin biosynthesis have been characterized. So far, neither genomic nor RNA-seq data have been available for the species. In the present study, we *de novo* sequenced two bilberry fruit developmental stages, unripe green (G) and ripening (R). A total of 57,919 unigenes were assembled of which 80.2% were annotated against six public protein databases. The transcriptome served as exploratory data to identify putative transcription factors related to fruit ripening. Differentially expressed genes (DEGs) between G and R stages were prominently upregulated in R stage with the functional annotation indicating their main roles in active metabolism and catalysis. The unigenes encoding putative ripening-related regulatory genes, including members of NAC, WRKY, LOB, ERF, ARF and ABI families, were analysed by qRT-PCR at five bilberry developmental stages. Our *de novo* transcriptome database contributes to the understanding of the regulatory network associated with the fruit ripening in bilberry and provides the first dataset for wild *Vaccinium* species acquired by NGS technology.

## Introduction

Bilberry (*Vaccinium myrtillus* L.) is a perennial dwarf shrub growing native at low temperature regions in northern hemispheres, most abundantly from the west coast of Northern Europe to Caucasus toward the northern Asia Pacific coast^1^. Bilberries are usually diploids, but clones with higher ploidy levels have been found among subspecies in North America. Bilberry is economically one of the most important wild berry species of genus *Vaccinium* in Europe, and has worldwide interest due to the high content of health-beneficial compounds^1^. Ripe bilberry fruits are characterized by dark blue pigmentation in both flesh and skin, indicating the presence of anthocyanin pigments, which have shown multiple benefits for human health^2^. The biosynthesis of anthocyanins and other flavonoid compounds is well understood and the key structural genes and transcription factors (TFs) controlling their biosynthesis have been characterized in many species. In bilberry, the expression of the structural and some regulatory genes has been studied in bilberry fruits but also in white- and pink-colored fruit representing rare but natural mutants of the species^3,4^. Our recent studies have shown that the anthocyanin profiles in *Vaccinium* spp. berries are regulated by prevalent light and temperature conditions^5–8^.

Fruit development and ripening are regulated by complex processes as a combination of internal and external cues. Fleshy fruits are physiologically defined as either climacteric or non-climacteric according to the differences in respiration rate and production of plant hormone ethylene at ripening. Ethylene production peaks at the initiation of ripening in climacteric fruits and ethylene is considered as the most important hormone controlling the ripening of climacteric fruits^9–11^, but has also been associated to ripening of some non-climacteric fruits^12^. In addition, abscisic acid (ABA) and auxin, which both play crucial roles in plant growth and development as well as environmental stress responses, have recently been proposed to have an important role in fleshy fruit ripening^13,14^. Especially, ABA has been associated in non-climacteric fruit ripening, also in bilberry^15^. The ripening process of non-climacteric bilberry includes morphological, biochemical and physiological modifications, particularly accumulation of anthocyanin pigments as well as changes in texture, taste and flavor as in many fleshy fruits^16,17^.

A number of regulatory genes belonging to different gene families have been proposed to control the developmental and ripening processes of fruits^17^. Particularly studies have been conducted in tomato, in which RIPENING INHIBITOR (RIN), COLORLESS NON-RIPENING (CNR), and NON-RIPENING (NOR) TFs are well characterized as key regulators in fleshy fruit ripening process^16,18,19^. Several MADS-box genes, i.e. AGAMOUS (AG), Tomato AGAMOUS-like 1 (TAGL1), and FRUITFUL (FUL), have been described playing redundant functions in tomato fruit development and ripening processes^17,20,21^. In addition, a role for WRKY and NAC TFs in fruit ripening has been suggested in addition to their roles in stress responses^22–24^. In bilberry, a more comprehensive understanding on the regulation of fruit ripening beyond anthocyanin biosynthesis still needs further investigations. However, neither the genomic nor the transcriptomic data of bilberry are currently available in public databases hampering these studies.

RNA sequencing (RNA-seq) technology is a viable option to study transcriptome by interpreting the transcript structure, single nucleotide polymorphism (SNP) information, discovering the candidate genes involved in various metabolic pathways and understanding complex biological processes under different conditions^25^. Deep sequencing by RNA-seq has mostly been carried out on cultivated plant species but less on wild species. Various transcriptome projects have been accomplished for cultivated *Vaccinium* berry species in order to identify the novel genes and regulators related to biosynthesis of bioactive metabolites, especially flavonoids^26–29^. Besides berries of genus *Vaccinium*, transcriptome analyses have also been performed e.g. for black raspberry (*Rubus* c*oreanus*)^30^, strawberry (*Fragaria* × *ananassa*)^31^, grapevine (*Vitis vinifera*)^32^ and Chinese bayberry (*Myrica rubra*)^33^.

In the present study, we applied the next generation sequencing (NGS) technology to establish *de novo* transcriptome assembly of wild *V. myrtillus* during fruit ripening. For constructing the libraries, we sequenced two developmental stages of bilberry fruit i.e. unripe green fruit (G) and ripening purple fruit (R)^34^. The RNA-seq dataset was used to identify candidate TFs related to bilberry fruit ripening and their expression was further studied in more detail during bilberry fruit development by qRT-PCR.

## Results and Discussion

### RNA-seq and *de novo* transcriptome assembly

The bilberry transcriptome at two different fruit developmental stages (G and R) were obtained by using the Ion Torrent platform. The application of Ion Torrent (Ion PGM^TM^ system) technology enabling a 400-base chemistry is a relevant choice (i.e. cost-effective, rapid platform with great output and long read lengths) to perform RNA sequencing for non-model organism under the absence of reference genome^35^. By using Ion Torrent system, approximately 3,3 million high quality reads from G and R libraries were generated for assembly in which adapters and ambiguous bases had been removed after sequencing. An overview of statistics is shown in Table 1. *De novo* assembly yielded 57,191 unigenes and N50 value, a standard measure of the assembly quality^36^, of 656 bp from the statistics of all transcripts (Table 1). In the size distribution for unigenes, the number of unigenes was inversely proportional to the length of unigenes (Supplementary Fig. S1). Overall, we considered that the quality of RNA-seq dataset was appropriate for the further analyses.

**Table 1:** Summary of the sequencing and the Trinity *de novo* assembly.

Ripening stage	Unripe Green (G)	Ripening (R)
After sequencing		
Total clean reads	1,656,566	1,720,492
After assembly of combined data		
Total number of unigenes	57,919	
Length of all unigenes (bp)	30,791,407	
GC content (%)	43.9	
Mean length of unigenes (bp)	532	
Unigene N50 (bp)	656	

### Sequence annotation and classification

Genome annotation is one of the first bioinformatics steps to identify new sequences related to biologically relevant function^36^. In this study, a total of 80.2% of the assembled bilberry unigenes were successfully annotated to at least one of the six considered public protein databases and 3.4% of the unigenes were assigned to all six databases (Table 2). The description of the bilberry unigenes was only given with top blast hits (Supplementary Table S1). Approximately 20% of unigenes were not annotated, which might be either due to non-coding regions or caused by the inadequate length of sequences or the lack of information in databases^37^. The non-coding regions may have information or insight functions in RNA regulatory network that need further investigations^37^. The bilberry *de novo* transcriptome with high annotation percentage provides a new valuable resource for gene level studies in *V. myrtillus* and facilitates the discovery of novel candidate genes involved in the metabolism and ripening process of bilberry fruits, and might also be useful for studies in closely related species^25^.

**Table 2:** Summary of the functional annotation of assembled bilberry unigenes with public protein databases using BlastX cut-off E-value of 1E-5.

	Number of unigenes	% Annotated unigenes
**Total number of assembled unigenes**	**57,919**	
Gene annotation against NR	39,854	68.8
Gene annotation against Swiss-Prot	32,217	55.6
Gene annotation against InterPro	37,534	64.8
Gene annotation against KOG	8,710	15.0
Gene annotation against KEGG	7,905	13.6
Gene annotation against GO	36,126	62.4
Unigenes matching all six databases	1,973	3.4
Total annotated unigenes	46,439	80.2

**Figure 1:**
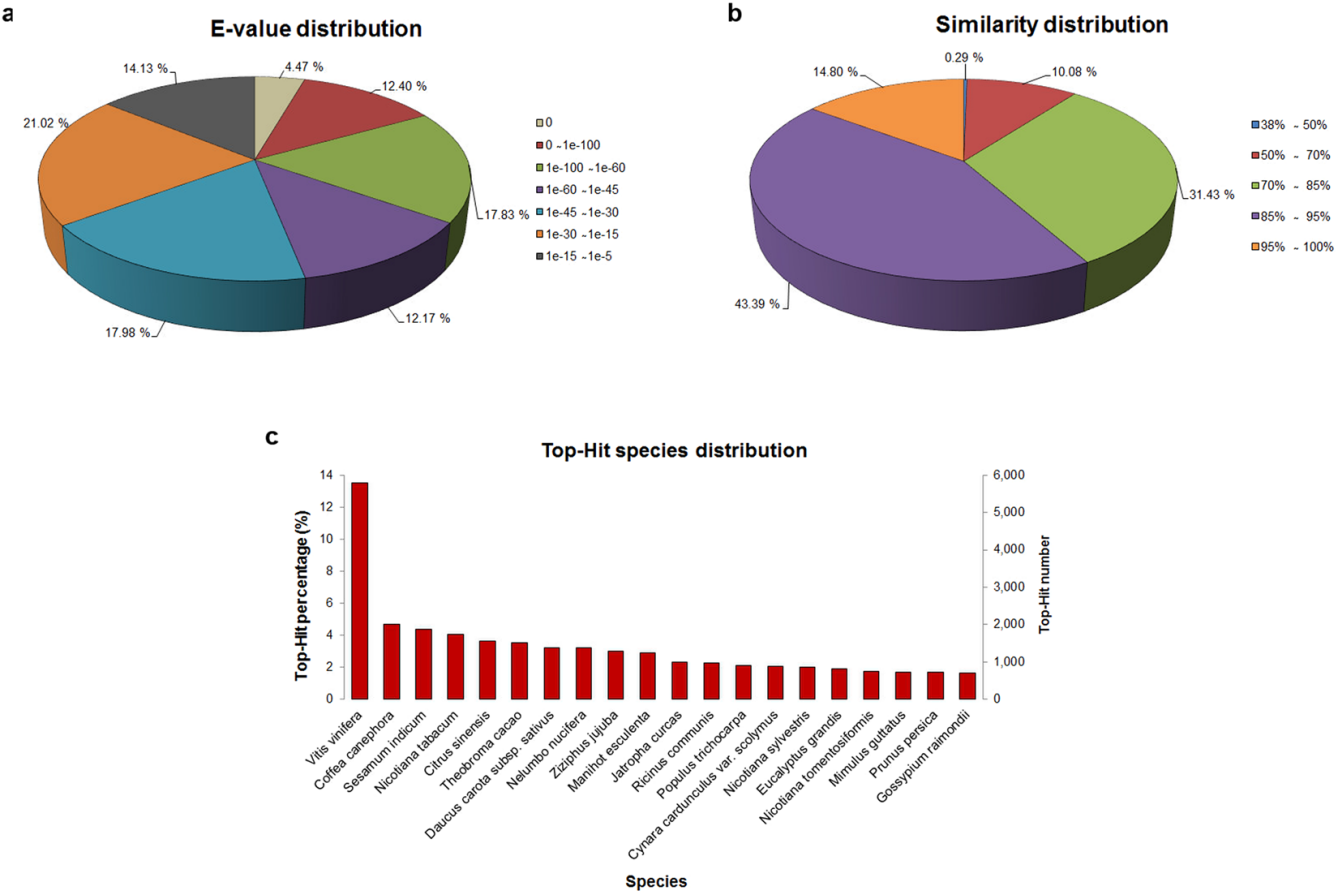
Distribution of bilberry unigenes annotated to the NCBI NR database using BlastX with cut-off E-value of 1E-5. (**a**) E-value distribution of annotated unigenes. (**b**) Sequence similarity distribution of annotated unigenes. (**c**) Species distribution of annotated unigenes matching the top 20 species. Bars represent the numbers of blast top-hit of bilberry unigenes in each species. Left Y-axis represents the percentages of blast top-hit and right Y-axis represents the numbers of blast top-hit.

The sequence homology search analysis indicates the reliability of blast result in the functional annotation study^38^. According to the E-value distribution, 46.9% of the annotated sequences showed very strong homology with E-value < 1E-45, and 53.1% showed strong homology with E-value from 1E-45 to 1E-5 (Fig. 1a). The distribution of sequence similarity with available sequences showed that 89.6% of the sequences had similarity higher than 70%, whereas only 10.4% of the sequences showed similarity ranging from 38% to 70% (Fig. 1b) i.e. suggesting a good match between the assembled and known sequences. The analysis of twenty top-hit species for the best match from each sequence showed that the annotated unigenes had the highest homology with sequences from *Vitis vinifera* (13.5%) followed by *Coffea canephora* (4.7%), *Sesamum indicum* (4.4%), *Nicotiana tabacum* (4%), and *Citrus sinensis* (3.6%) (Fig. 1c).

**Figure 2:**
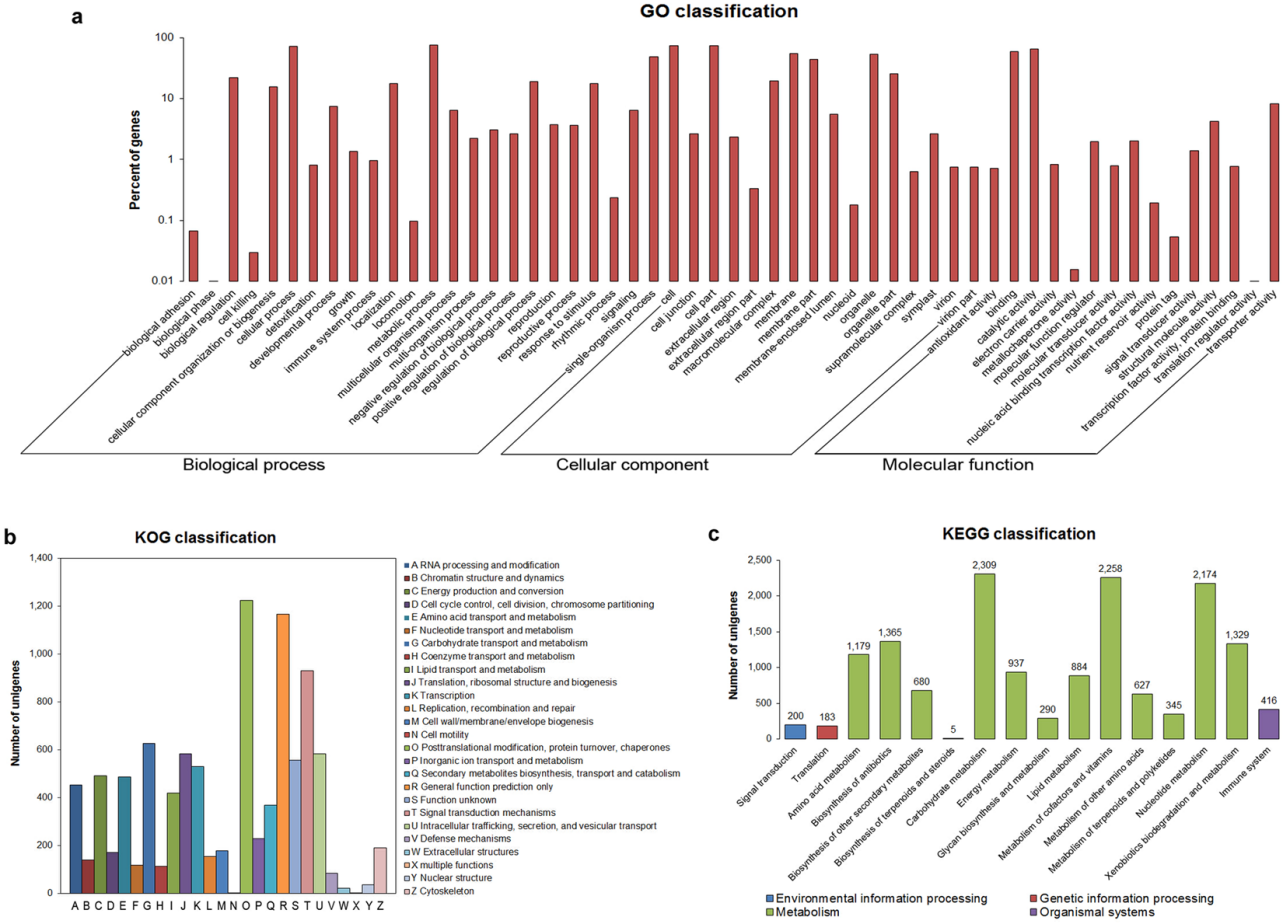
Functional classification of bilberry transcriptome. (**a**) GO classification. Bars represent the percentage of unigenes assigned into 55 GO sub-categories of three main categories: Biological process, Cellular component, Molecular function. (**b**) KOG classification. Bars represent the numbers of unigenes assigned into 26 KOG classes. (**c**) KEGG classification. Bars represent the numbers of unigenes clustered into four KEGG Orthology (KO) hierarchies. Numbers on the top of bars indicate the number of unigenes in each KO group.

In the present study, 62.4% of all unigenes were assigned into 55 functional groups belonging to three main GO categories, i.e. Biological process, Cellular component and Molecular function (Fig. 2a, Supplementary Table S2). Under the Biological process category, the predominant portion of unigenes represented “metabolic process”, “cellular process” and “single-organism process”. Within the Cellular component category, “cell” and “cell part” were the most abundant sub-categories, followed by “membrane”, “organelle”, “membrane part”, and “organelle part”. In the Molecular function category, “catalytic activity” and “binding” sub-categories constituted the major proportion of unigenes. In the KOG analysis, 8,710 unigenes were categorized into 26 classes (Fig. 2b). Among them, “posttranslational modification, protein turnover, chaperones”, “general function prediction only”, and “signal transduction mechanism” represented three of the largest classes, accounting for 14%, 13% and 11% of KOG annotated unigenes, respectively. In the KEGG pathway analysis, a total 7,905 unigenes were mapped onto 149 biological pathways clustered into four KEGG Orthology (KO) hierarchies of functional ortholog system (Supplementary Table S3). Among those KO hierarchies, a total of 97% of the KEGG annotated unigenes belonged to Metabolism with 13 KO groups (Fig. 2c). The largest KO group was “carbohydrate metabolism”, followed by “metabolism of cofactors and vitamins” and “nucleotide metabolism”. We identified 680 unigenes (9% of KO hierarchy “Metabolism”) associated in “biosynthesis of other secondary metabolites”, which were assigned to 21 pathways (Supplementary Table S3). Among these pathways, “phenylpropanoid biosynthesis” pathway was found to be the most abundant assignment with 297 unigenes followed by “flavonoid biosynthesis” with 105 unigenes, “flavone and flavonol biosynthesis” with 21 unigenes, and “anthocyanin biosynthesis” with 13 unigenes. These functional analyses emphasized the key metabolic activities turned on during bilberry ripening.

### DEGs between G and R stages

**Figure 3:**
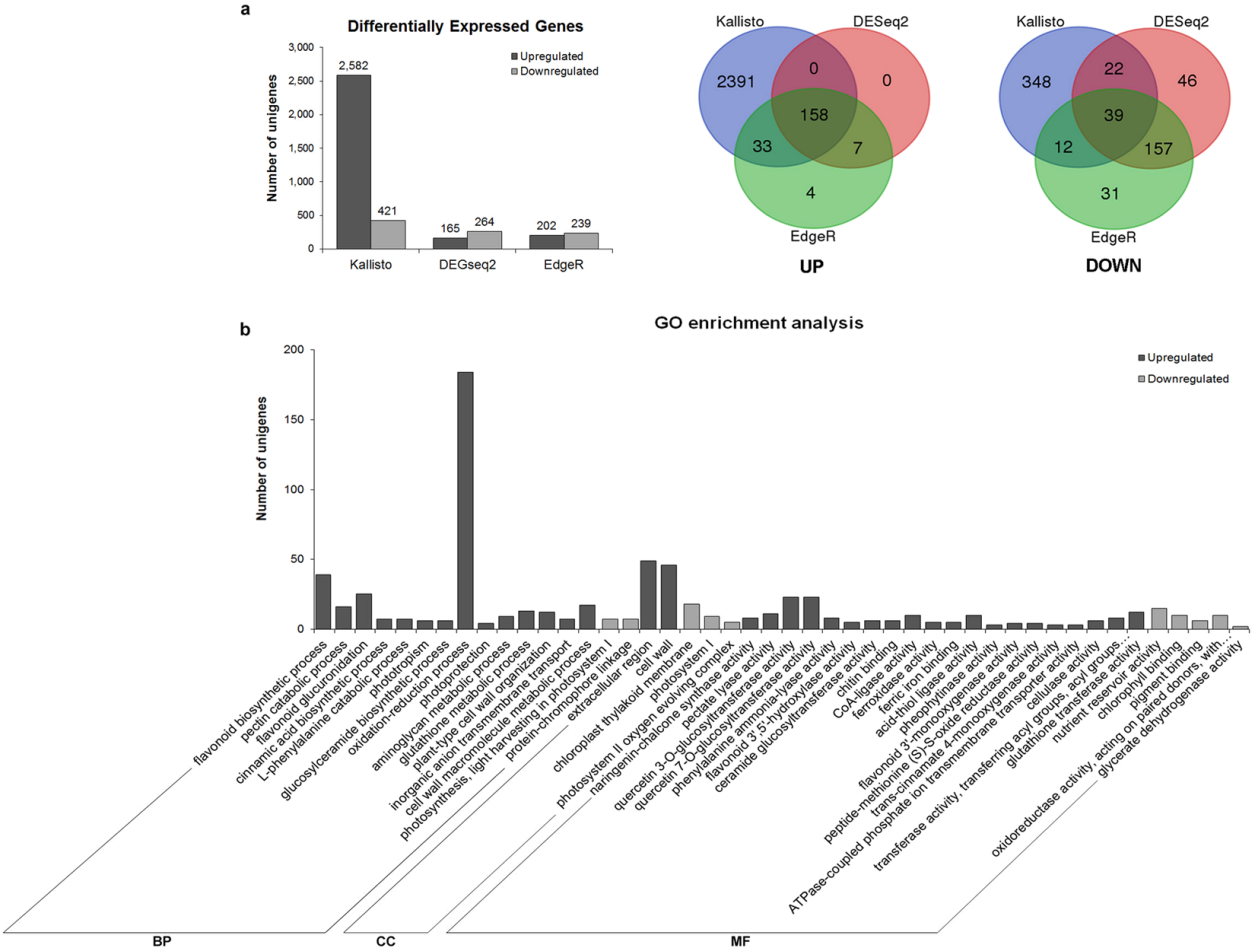
Differentially expressed genes (DEGs) analysis between G and R stages. (**a**) Number of upregulated and downregulated unigenes in R stage compared to G stage were identified by three methods Kallisto, DESeq2, and EdgeR. Bars represent the numbers of bilberry unigenes identified as DEGs. Numbers on the top of bars indicate the number of upregulated and downregulated DEGs in R stage compared to G stage, respectively. G and R indicate two bilberry developmental stages which are used for transcriptome libraries. Venn diagrams represent the comparison of up- and downregulated DEGs among Kallisto, DESeq2, EdgeR methods (**b**) GO enrichment analysis of bilberry DEGs. Bars represent the numbers of up- and downregulated DEGs in R stage compared to G stage which were assigned into 37 over-represented GO terms (p-value < 0.05) of three main categories: BP = Biological process, CC = Cellular component, MF = Molecular function.

As the first transcriptome level study in bilberry, we used three different software packages and pipelines for the detection of differential gene expression between two fruit ripening stages. From our dataset, a total of 3,428 unigenes were determined as differentially expressed genes (DEGs) between G and R of bilberry. Of these, 3,003 DEGs were found from Kallisto pipeline which is higher than the DEGs identified with DESeq2 (429 unigenes) and EdgeR (441 unigenes) (Fig. 3a). Of all DEGs, 197 unigenes were found among all three packages while 22, 45 and 164 unigenes were overlapping in pairwise comparison between methods Kallisto-DESeq2, Kallisto-EdgeR, and DESeq2-EdgeR, respectively. The results show that more downregulated genes were detected compared to upregulated genes with EdgeR and DESeq2 (Fig. 3a). Vice versa in Kallisto pipeline, the upregulated DEGs were more predominant than downregulated ones in R stage compared to G stage (Fig. 3a). In the present study, this transcriptomic dataset was used as an exploratory data to identify candidate transcription factors (TFs) in bilberry fruit ripening for further analysis by qRT-PCR.

GO enrichment analysis was carried out to provide functional classification for DEGs (Supplementary Table S4). Among the DEGs that were upregulated at R stage, 36 sub-categories of three main GO categories were found to be enriched (Fig. 3b). The Biological process category had 14 over-represented GO terms. From them, a high number of upregulated DEGs were found under terms “oxidation-reduction process”, “flavonoid biosynthesis process”, and “flavonoid glucuronidation”. Molecular function category, which had highest number of over-represented GO terms (20 sub-categories), exhibited DEGs most abundantly in “quercetin 3-O-glucosyltransferase activity”, “quercetin 7-O-glucosyltransferase activity”, and “glutathione transferase activity” sub-categories. There were two sub-categories belonging Cellular component category showing highly enriched in “extracellular region” and “cell wall”. In the group of downregulated DEGs, 10 sub-categories were identified as enrichment of GO terms in photosynthesis processes including photosynthetic light-harvesting in the Biological process, chlorophyll/pigment binding protein, and enzyme activities in the Molecular function, and thylakoid membrane as well as photosystem complexes in the Cellular component (Fig. 3b, Supplementary Table S4). The GO enrichment analyses suggest the important functions of the up- and downregulated DEG groups corresponding to developmental processes during bilberry fruit ripening, especially the photosynthesis at green stage (G) and flavonoid biosynthesis at ripening purple stage (R). Following the fact that the ripening process of fleshy fruits includes major metabolic and structural changes such as accumulation of pigments, especially anthocyanins in case of bilberry^6^, and fruit softening due to modification of cell wall^16,17^, our results imply that the bilberry transcriptome associates with these complex processes common to fruits during ripening.

### The structural genes in flavonoid pathway at G and R stages

The flavonoid pathway is well studied with key genes identified in many flowering plants and fruit species^39^. In this study, the homologs of the structural genes involved in flavonoid pathway were identified from bilberry transcriptome database. A total of 54 unigenes encoding 15 enzymes of the flavonoid biosynthesis were identified as DEGs between G and R developmental stages of bilberry fruit (Supplementary Table S5). There were more than one unigene clustered into one enzyme, which may represent different fragments of a transcript or different isoforms or alleles of the same enzyme. The flavonoid pathway genes homologs encoding chalcone synthase (CHS; c12755_g3_i2, c12904_g2_i1), anthocyanidin synthase (ANS; c12683_g5_i4, c11284_g1_i1), and UDP-glucose flavonoid glucosyltransferase (UFGT; c12490_g1_i1), showed high expression at R stage compared to G stage (Supplementary Table S5). The expression levels of these genes were analysed by qRT-PCR in five developmental stages of bilberry fruit (Supplementary Fig. S2) demonstrating the increase in the expression at the onset of fruit ripening in accordance with our previous report^3^. Notably, UFGT, which catalyzes the last step of anthocyanin biosynthesis was found in qRT-PCR analysis to be highly upregulated at stage R, indicating the important role of the gene in during bilberry fruit ripening. Our results are consistent with the earlier studies showing upregulation of the structural anthocyanin pathway genes simultaneously with the visible accumulation of the anthocyanin pigments^40^.

### Identification of candidate TFs related to fruit ripening

From DEGs data, we especially focused on identifying the candidate transcription factors (TFs) involved in bilberry fruit ripening process that were then studied in more detail by qRT-PCR analysis. Altogether 33 TF genes belonging to different gene families showed marked difference in their expression between the two developmental stages of fruit ripening in bilberry RNA-seq dataset (Supplementary Table S6).

**Figure 4:**
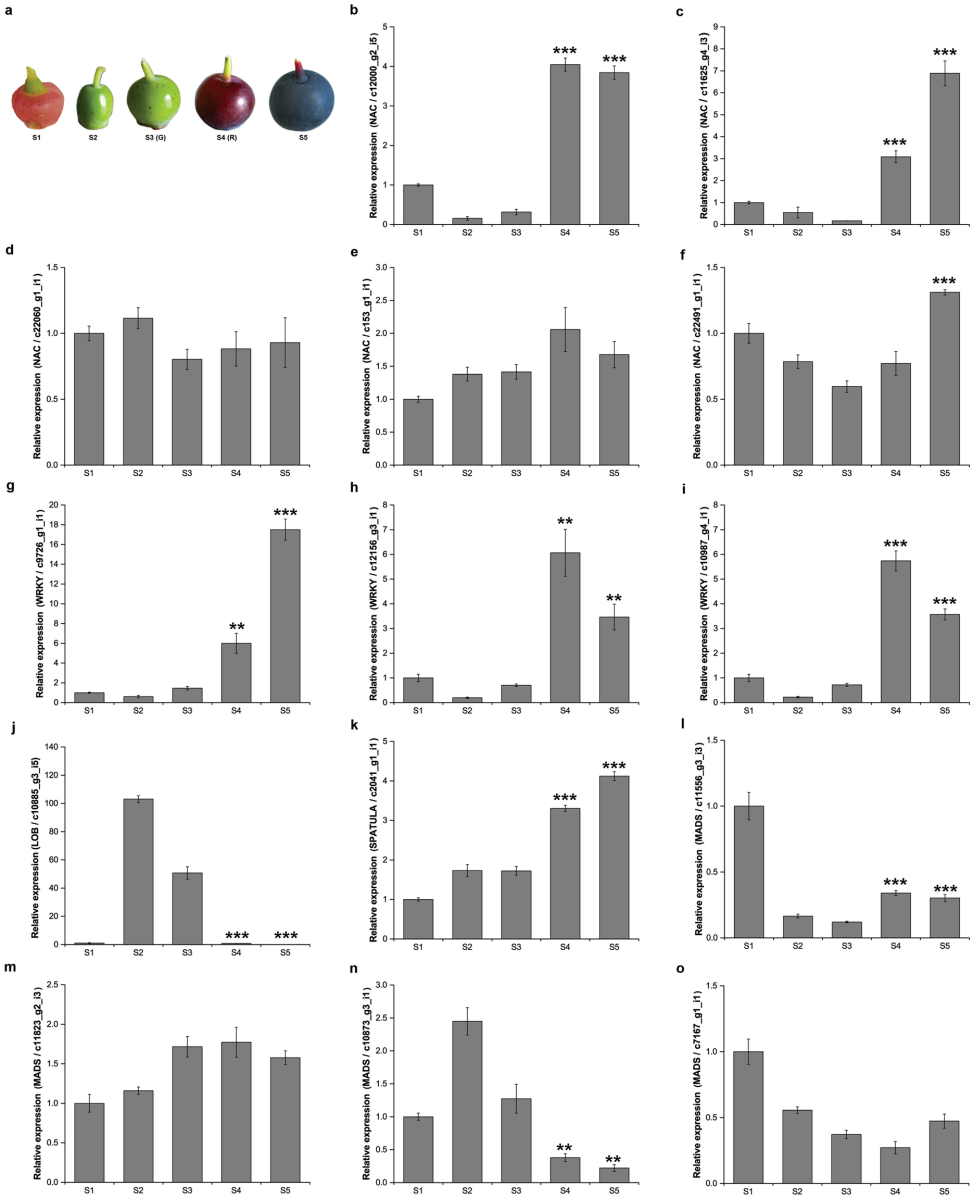
qRT-PCR analysis of TFs during bilberry fruit development. (**a**) Five development stages of fruit during ripening process. S1–Flower, S2–Small unripe green fruit, S3–Large unripe green fruit (G), S4–Ripening purple fruit (R), S5–Fully ripe blue fruit. G and R indicated two bilberry stages which are used for construction of transcriptome libraries. Relative expression of (**b**–**f**) NAC, (**g–i)** WRKY, (**j**) LOB = Lateral Organ Boundaries domain, (**k**) SPATULA, (**i–o**) MADS. Bars represent the relative expression levels of unigenes in each stage normalized with respect to the internal control *GAPDH*. Error bars represent standard error of four biological replicates. *Asterisks* indicate significant differences between early stage (S3) and ripening stages (S4, S5) at level ^*^p < 0.05, ^**^p < 0.01, ^***^p < 0.001 using Student’s *t*-Test.

NAC gene family has recently been suggested to be a regulator in fruit ripening^16,24^. NOR is the best known member of NAC family operating upstream of RIN and regulating ethylene-related gene expression in tomato ripening process^41^. In the present study, a total of six unigenes encoding NAC TFs were identified as DEGs, of which five were upregulated and one unigene (c3865_g1_i1) was downregulated. According to qRT-PCR analysis performed at five bilberry developmental stages (Fig. 4a), all the identified five upregulated members of NAC family were verified to be upregulated at the onset of fruit ripening, although at different levels (Fig. 4b–f). Especially, two NACs (c12000_g2_i5 and c11625_g4_i3), which had more than 60% identity at amino acid level with *LeNOR* (Supplementary Table S6), showed significant upregulation in their expression at the onset of fruit ripening in qRT-PCR analysis (Fig. 4b,c). Considering the important role of NOR gene in tomato, these two NACs may also have a regulatory role in bilberry fruit ripening. Moreover, NAC gene have also been reported as activators for anthocyanin biosynthesis in Arabidopsis plants under high-light stress (*ANAC078*)^42^ as well as in maturing peach fruits (*PpNAC*1)^43^. Also, *SlNAC4* has been reported as a component in fruit ripening and carotenoid metabolism network in tomato^24^. Thus, we infer that bilberry NACs could possibly be involved in regulation of the flavonoid biosynthesis network during fruit ripening, for which a more detailed study needs to be carried out in the future.

In non-climacteric pepper, *CaWRKY* gene family associated in regulatory network in fruit ripening was recently reported^23^. In bilberry transcriptome, four WRKY unigenes were identified as DEGs. The relative expression of three WRKYs significantly increased at fruit ripening according to qRT-PCR results (Fig. 4g–i), indicating that these WRKYs may have a regulatory role in ripening of the non-climacteric bilberry fruit. Especially, the qRT-PCR results showed that two unigenes (c12156 and c10987) were highly upregulated at the onset of fruit ripening (Fig. 4h,i) while one unigene (c9762_g1_i1) was expressed most prominently at the fully ripe stage implying a dominant role of this gene in ripe, rather than ripening berry (Fig. 4g).

There are only a few studies on the potential role of plant-specific gene family Lateral Organ Boundaries domain (LOB) in the regulation of fruit ripening processes^44,45^, and so far, the knowledge on molecular mechanism of LOB TFs in fruit development and ripening remains unclear. In the present study, we identified four LOB unigenes being downregulated at R ripening stage. One LOB (c10885_g3_i5) was significantly downregulated at the ripening stage in our qRT-PCR analysis (Fig. 4j). The amino acid sequence of this gene corresponds to *AtLBD39* (67.9% identity; Supplementary Table S6), which was previously reported as a negative regulator of anthocyanin biosynthetic pathway in Arabidopsis^44^. Moreover, Ba *et al*. reported that the LOB TF members, *MaLBDs*, were ethylene-induced regulators controlling banana ripening and acting as transcriptional activators for EXPANSIN in cell wall modification^45^. These earlier reports, in addition to the present qRT-PCR expression data, suggest that bilberry LOB candidate gene may have a role in the early fruit development or serve as a negative regulator of bilberry fruit ripening.

From our dataset, we found only one DEG (c2041_g1_i1) annotated as SPATULA having 100% identity to *Vaccinium corymbosum VcbHLH032* (Supplementary Table S6). The transcripts of this unigene were verified by qRT-PCR to be significantly increased at fruit ripening compared to early fruit development (Fig. 4k). The function of SPATULA, an ortholog of ALCATRAZ in Arabidopsis, controls fruit patterning and early fruit development^17,46^. SPATULA belongs to the bHLH superfamily, which is well characterized in various functions including regulation of flavonoid biosynthesis in different species^47–50^. However, so far there has been no report on the involvement of the SPATULA in ripening process of fleshy fruits. According to our findings, bilberry SPATULA gene can contribute to metabolic activities in bilberry fruit ripening and deserves further investigations in the future.

In tomato, many MADS-box genes have shown to be essentially involved in ripening regulatory network^18–21^. In DEGs analysis of bilberry transcriptome, five MADS-box TFs showed upregulation at R stage compared to G stage of which one unigene (c12746_g6_i2) was identified as *VmTDR4*^4^ (Supplementary Table S6). In our analysis of expression by qRT-PCR, two MADS genes were only slightly upregulated at bilberry fruit ripening (Fig. 4l,m). The MADS gene (c10873_g3_i1) identified as AGAMOUS (Supplementary Table S6) was expressed at high level in the S2 stage of bilberry fruit development (Fig. 4n). It can be speculated that the AGAMOUS is possibly involved in regulation of early stages of fruit development due to its redundant functions in flower and fruit development regulation in previous studies^17,51^. Furthermore, one unigene potentially encoding SQUAMOSA TF (c7167_g1_i1) had 63% identity with *Camellia sinensis* SQUAMOSA promoter binding protein like 6 (SPL6; accession number AOO19736.1) (Supplementary Table S6). However, based on qRT-PCR results, its expression level did not change markedly during ripening of bilberry fruit (Fig. 4o).

### Identification of hormone-related TFs associated in fruit ripening

In the present study, we identified 13 candidate unigenes designed as DEGs between G and R developmental stages potentially encoding TFs that are related to plant hormone-mediated fruit ripening regulation (Supplementary Table S6) to be further studied by qRT-PCR.

**Figure 5:**
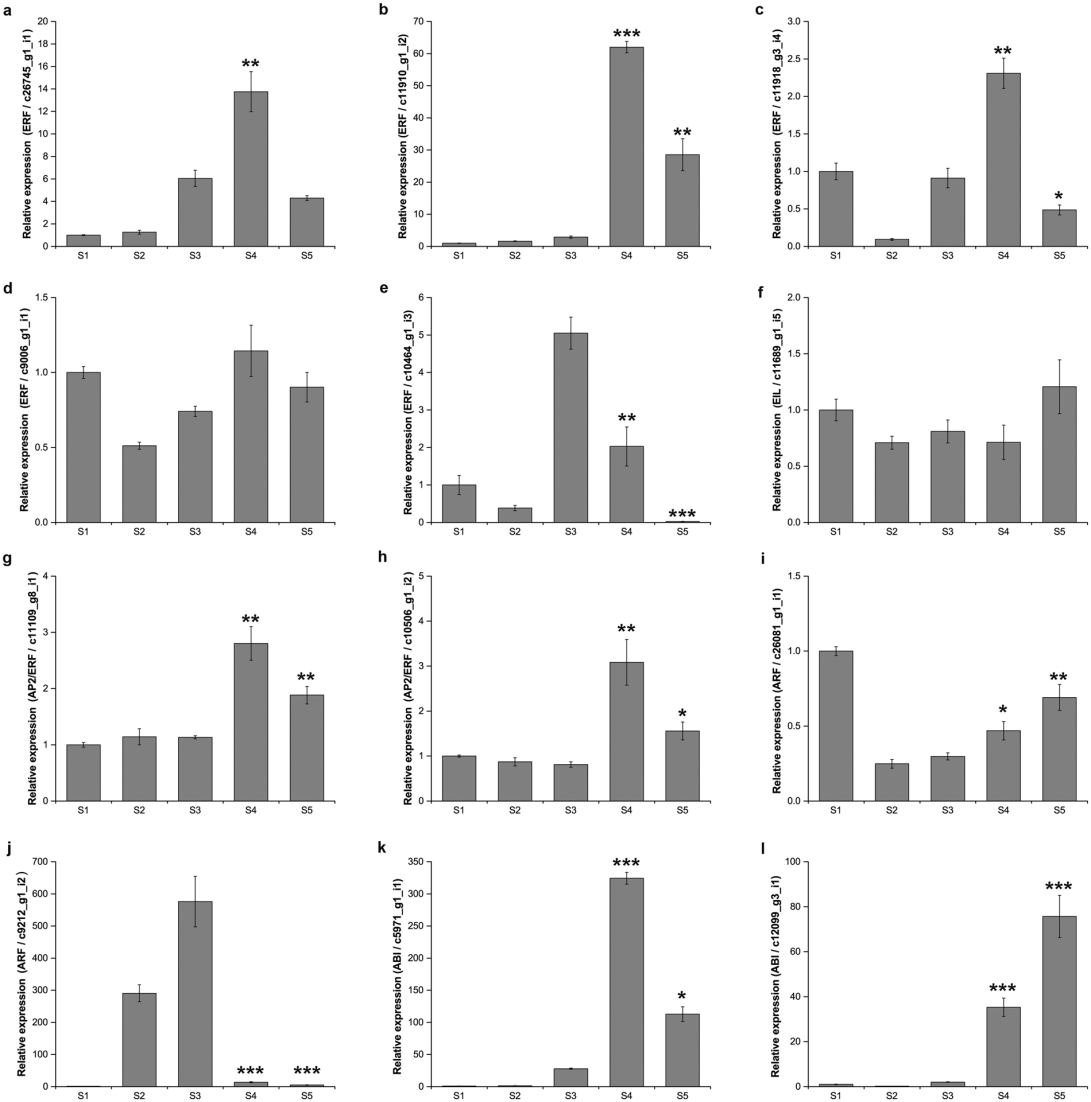
qRT-PCR analysis of hormone-related TFs during bilberry fruit development. (**a–e**) ERF = Ethylene Responsive Transcription Factor, (**f**) EIL = Ethylene Insensitive like, (**g**,**h**) AP2/ERF = APETALA2/ Ethylene Responsive transcription Factor, (**i,j**) ARF = Auxin Response Factor, (**k,l**) ABI = Abscisic acid Insensitive. Bars represent the relative expression levels of unigenes in each stage normalized with respect to the internal control *GAPDH*. Error bars represent standard error of four biological replicates. S1–Flower, S2–Small unripe green fruit, S3–Large unripe green fruit (G), S4–Ripening purple fruit (R), S5–Fully ripe blue fruit. G and R indicated two bilberry stages which are used for construction of transcriptome libraries. *Asterisks* indicate significant differences between early stage (S3) and ripening stages (S4, S5) at level ^*^p < 0.05, ^**^p < 0.01, ^***^p < 0.001 using Student’s *t*-Test.

The ripening of climacteric fruits differentiates from non-climacteric fruits by the presence of increasing respiration and ethylene biosynthesis at ripening stage. Hence, ethylene is considered a critical factor in the climacteric fruit ripening and has been intensively studied for decades^9–11^. The genes regulated by ethylene have been found to have different roles in tomato ripening, i.e. *LeERF1* acts as a positive mediator for ethylene signaling, while *SlERF6* is a negative regulator in carotenoid biosynthetic pathway^52,53^. In non-climacteric pepper fruit, Lee *et al*. discovered that *EIL*-like genes could induce fruit ripening by acting as a downstream component of ethylene-mediated signaling^12^. In this study, we identified from our dataset six ethylene-responsive transcription factor genes (ERF) as DEGs. Three ERFs (c26745_g1_i1, c11910_g1_i2, and c11918_g3_i4) were significantly upregulated at R stage in qRT-PCR analysis (Fig. 5a–c). Especially, unigene c11910_g1_i2 sharing 63% identity at amino acid level with *LeERF1* (Supplementary Table S6) was highly expressed at the onset of fruit ripening suggesting that the gene may act as a positive regulator in bilberry fruit ripening. On the other hand, the expression of one ERF (c9006_g1_i1) was not much different during five bilberry development stages based on qRT-PCR analysis. Instead, another ERF (c10464_g1_i3), which exhibited decreasing expression at fruit ripening (Fig. 5d,e), may act as a negative regulator of bilberry fruit ripening. Additionally, we found one DEG encoding EIL having 51% identity with *SlEIL* (Supplementary Table S6). However, its expression did not vary significantly during bilberry fruit development (Fig. 5f). Also, two AP2/ERF genes, which belong to a TF family that was characterized as an important regulator of tomato fruit ripening^54^, were designated as DEGs and their expressions were verified by qRT-PCR to be upregulated at the onset of fruit ripening (Fig. 5g,h).

In the past few years, the role of auxins has also been discovered as negative regulators of fruit ripening^55,56^. In particular, *SlARF4* plays redundant functions in tomato, such as acting as a negative regulator of starch biosynthesis, controlling chlorophyll accumulation and sugar metabolism in tomato fruit development^56^. Another auxin related gene, *ARF106*, was found to regulate fruit size in apple^55^. Auxin has also been proposed to control the receptacle cell expansion during early stages of strawberry fruit development^14^. In our DEG data, we identified two Auxin Response Factor genes (ARF) of which one unigene (c26081_g1_i1) was upregulated and another (c9212_g1_i2) downregulated at stage R compared to stage G that was also verified by qRT-PCR analysis (Fig. 5i,j). The significant down-regulation of ARF gene (c9212_g1_i2) suggests that this gene may have a role as a negative regulator of bilberry fruit ripening.

In non-climacteric fruits such as bilberry, ABA has been shown to control the regulation of ripening and anthocyanin biosynthesis^57,58^. Medina-Puche *et al*. showed that ABA could activate the expression of *FaMYB10* controlling the strawberry ripening process and anthocyanin biosynthesis^14,59^. In our study, two unigenes (c5971_g1_i1 and c12099_g3_i1) that were identified to encode Abscisic Acid Insensitive (ABI) were significantly upregulated at the onset of fruit ripening as verified by qRT-PCR analysis (Fig. 5k,l). These two ABI genes showed high homology to grapevine *VvABF2* an important ABA-dependent regulator in grape fruit ripening^58^ (Supplementary Table S6). It is also important to note that our previous studies have shown the ABA biosynthesis to be induced at the onset of fruit ripening in bilberry indicating role of ABA in bilberry fruit ripening^15^. These results imply that the ABI genes may act as the main TFs associated with the ABA-regulated fruit ripening also in bilberry.

## Conclusions

The described *de novo* transcriptome assembly provides the first RNA-seq data for bilberry. Our annotated dataset identified a large number of unigenes associated to metabolic and catalytic activities. Especially, we identified through DEGs analyses many candidate TFs of fruit ripening belonging to NAC, WRKY and LOB gene families as well as TFs related to signaling by plant hormones such as ethylene, auxin, and ABA. Based on our qRT-PCR analysis, some of these TFs may play important roles in bilberry fruit ripening, and will be subjected to more detailed functional studies in the future. Overall, the findings of the present study offer new insights into regulatory network of fruit ripening process in bilberry and, more generally, valuable sequence information for further studies and applications in wild *Vaccinium* species.

## Materials and Methods

### Plant material

Bilberry (*Vaccinium myrtillus* L.) fruits were collected from natural forest stand in Oulu (65°01′N, 25°28′E), Finland. Fruits were harvested from June to August at five developmental stages as described previously^34^: S1. Flower (June 7^th^, anthesis), S2. Small unripe green fruit (15 days after anthesis), S3. Large unripe green fruit (G) (28 days after anthesis), S4. Ripening purple fruits (R) (34 days after anthesis), S5. Fully ripe blue fruit (55 days after anthesis) (Fig. 4a). Bilberry samples were collected at the same time for the construction of transcriptome library and qRT-PCR analyses. For *de novo* transcriptome analysis, approximately 15–25 berries depending on fruit size at two stages, S3 and S4 (about 3 grams of berries), were utilized for RNA sequencing. For qRT-PCR analysis, four biological replicates of each ripening stage were used with each replicate having approximately 9–12 berries (about 2 grams) depending on fruit size. Immediately after collection, all samples were frozen in liquid nitrogen and stored at −80 °C until used for RNA extraction.

### Transcriptome sequencing

Bilberry mRNA from fruits at developmental stage S3 (G) and stage S4 (R) was separately extracted for transcriptome sequencing. Briefly, frozen fruit tissues were ground to fine powder under liquid nitrogen using mortar and pestle. The initial incubation with RNA extraction buffer and extraction with chloroform:IAA was conducted as previously described^60^. The extract was further incubated with Dynabeads Oligo (dt)_25_ (ThermoFisher Scientific) and LiCl (670 mM) at +4 °C for 75 min. After incubation, the beads were washed on a magnet according to the manufacturer’s instructions. To elute mRNA, the beads were incubated with molecular grade water at + 78 °C for 2 min, and the supernatant collected on a magnet. The mRNA was stored at −80 °C until used within a day for preparation of transcriptome libraries.

To create the *de novo* transcriptome assembly, the Ion Torren platform was employed for RNA sequencing. Concentration, purity and intactness of mRNA were verified by Bioanalyzer using RNA Pico chip (Agilent Technologies). Libraries were prepared with Ion Total RNA-Seq Kit v2 (ThermoFisher Scientific). For RNAse III fragmentation step, enzyme was diluted 1:10 in reaction buffer in order to get longer distribution of fragmented mRNA molecules. In subsequent steps, manufacturer’s instructions were followed. Libraries were verified for quality and concentration by Bioanalyzer using High-Sensitivity DNA chip (Agilent Technologies). Sequencing of libraries was performed at Biocenter Oulu Sequencing Center (University of Oulu, Oulu, Finland) with Ion Torrent PGM sequencer using Ion Template OT2 400 kit, Ion PGM Sequencing 400 kit and Ion 318 v2 chip. All raw reads obtained in this study are available at GenBank Sequence Read Archive (SRA) under accession number SRX3387852 for G and SRX3387853 for R from BioProject ID PRJNA417893 (https://www.ncbi.nlm.nih.gov/bioproject/417893).

### *De novo* transcriptome assembly and functional annotation

After sequencing by Ion Torrent system, the adapters were automatically removed from the reads. Remaining reads were then assessed for high quality with FASTQC program based on three criteria: without adapters, ambiguous bases (Ns) contents, and with high sequence quality score. *De novo* transcriptome assembly was carried out using Trinity software r20140413p1, which is composed of three modules: Inchworm, Chrysalis and Butterfly based on de Bruijn Graph approach^61^. In undertaking the first analysis, reads from the two libraries were combined before running the program. Following processes included two phases: (1) clustering and building k-mer catalogs from RNA reads and linear contigs construction from k-mers (default value = 25) by Inchworm; (2) clustering and constructing de Bruijn graph for Inchworm contigs using Chrysalis and finally reconstructing individual graphs in parallel and resolving alternatively spliced isoforms and paralogous transcripts by Butterfly.

To investigate the potential functions of bilberry transcripts, all unigenes were annotated by BLASTX with an E-value threshold of 10^−5^ against six public protein databases: NCBI non-redundant protein (NR), Swiss-Prot, InterPro, euKaryotic Ortholog Groups of proteins (KOG), Kyoto Encyclopedia of Genes and Genomes (KEGG) Orthology (KO), and Gene Ontology (GO). Blast2GO program v4.0 was used to obtain NR, SwissProt, InterPro, GO, and KEGG annotations^62^. WebMGA was used in KOG annotation based on rpsblas 2.2.15 program against NCBI KOG database released 2/2/2011^63^. The threshold of E-value < 10^−5^ and 30% identity were used to infer the homology of two sequences^38^.

### Differentially expressed genes (DEGs) and GO enrichment

To identify changes in gene expression levels between the two bilberry fruit developmental stages, G and R, all assembled unigenes were calculated and normalized by using three different software packages and pipelines: (1) Kallisto v0.42.3 (https://pachterlab.github.io/kallisto/) was used to calculate bilberry transcript abundance to TPM value (Transcript count per million transcripts)^64^. The differential gene expression between G and R stages were determined based on an absolute value of log fold-change > 2.3 and a threshold of TPM count > 20. We also utilized (2) DESeq2 package running in R^65^ and (3) EdgeR package^66^ running in Chipster software^67^. To calculate gene expression using these two packages, we first used Chipster software for remapping the reads to the assembled transcriptome by using HISAT2^68^, then Cuffllink was used to assemble transcripts^69^, finally the aligned reads were counted by HTSeq^70^. When using DESeq2 and EdgeR programs, the differential expression of genes (DEGs) were analyzed with the application of FDR (False Discovery Rate) for adjusted P-value < 0.05. The differential gene expression level between the two stages was screened with absolute value of log fold-changes > 2 and the P-value < 0.1 from DESeq2 and < 0.01 from EdgeR.

GO enrichment analysis was performed to provide functional information for DEGs. Blast2GO program v4.0 was used to analyze all DEGs data (up- and downregulated genes) using Fisher’s Exact Test with multiple testing correction of FDR threshold 0.05 based on Benjamini Hochberg method^62^. The group of genes with P-value cut-off 5% was identified as enrichment (over-representation) of GO terms.

### qRT-PCR analysis

For the qRT-PCR analysis, total RNA was isolated from five different developmental stages of bilberry fruits with four biological replicates using method described previously^60^. The cDNA was synthesized from 5 µg of total RNA using Superscript III Reverse Transcriptase (Invitrogen) following manufacturer’s instructions. The cDNA was purified from genomic DNA with the method described previously^71^. The qRT-PCR analyses were performed with a LightCycler 480 instrument and software v1.5.0.39 (Roche Applied Sciences). The transcript abundance was detected using a SsoAdvanced Universal SYBR Green Supermix (Bio-Rad) with 15 µl total reaction volume. The PCR conditions were 95 °C for 10 min followed by 40 cycles of 95 °C for 10 s, 60 °C for 10 s and 72 °C for 20 s. Sequences of primers are listed in Supplementary Table S7. Glyceraldehyde-3-phosphate dehydrogenase (*GAPDH;* GenBank accession no. AY123769) was used as an internal control for the measurement of relative amount of transcripts. The results were calculated with LightCycler^®^ 480 software (Roche), using the calibrator-normalized PCR efficiency-corrected method (Technical note no. LC 13/2001, Roche). The amplification of only one product in qRT-PCR was confirmed by a melting curve analysis.

### Statistical analyses

Quantitative results of the gene expression analyses are presented in terms of means and standard errors (SE) for four biological replicates. Statistically significant differences between early stage S3 and ripening stages S4 and S5 at p-value < 0.1%, 1% and 5% were analyzed by Student’s *t*-Test using IBM SPSS Statistics program v25.

### Data availability

The data analyzed during the current study are included within the article and its addtional files. All unigene sequences from bilberry were deposited to GenBank Sequence Read Archive (SRA) under accession number SRX3387852 for G and SRX3387853 for R. The obtained sequences supporting the results of this article were deposited from NCBI database and their accession number are shown in Supplementary Table S6.

## Electronic supplementary material

Supplementary Information

Supplementary Table S1
