# Supplementary material for: Recognition of candidate transcription factors related to bilberry fruit ripening by de novo transcriptome and qRT-PCR analyses
Source: Sci Rep. 2018 Jul 2;8:9943. doi: 10.1038/s41598-018-28158-7 (PMC6028583; doi:10.1038/s41598-018-28158-7)
Supplement: Supplementary file 1 — Supplementary Information [file 41598_2018_28158_MOESM1_ESM.docx]

**Supplemental Information**

**Recognition of candidate transcription factors related to bilberry fruit ripening by *de novo* transcriptome and qRT-PCR analyses**

Nga Nguyen^1^, Marko Suokas^1,2^, Katja Karppinen^1^, Jaana Vuosku^1^, Laura Jaakola^3,4^, and Hely Häggman^1,*^

^1^Department of Ecology and Genetics, University of Oulu, FI-90014 Oulu, Finland

^2^Biocenter Oulu, University of Oulu, FI-90014 Oulu, Oulu, Finland

^3^Climate laboratory Holt, Department of Arctic and Marine Biology, UiT the Arctic University of Norway, NO-9037 Tromsø, Norway

^4^NIBIO, Norwegian Institute of Bioeconomy Research, NO-1431 Ås, Norway

*Correspondence: [hely.haggman@oulu.fi](mailto:hely.haggman@oulu.fi)

**The number of figures: 2**

**The number of tables: 7**

**Supplementary Table S1.** Blast top-hits from NCBI NR database for unigene sequences with cut-off E-value of 1E-5.

**Supplementary Table S2.** Statistics of Gene Ontology (GO) classification of bilberry transcriptome.

**Supplementary Table S3.** Statistics of KEGG orthology (KO) classification of bilberry transcriptome.

**Supplementary Table S4.** GO enrichment analysis of bilberry DEGs. The numbers of up- and downregulated DEGs in R stage compared to G stage which were assigned into 46 over-represented GO terms (p-value < 0.05) of three main categories: BP, Biological process; CC, Cellular component; MF, Molecular function.

**Supplementary Table S5.** DEGs analysis of flavonoid genes in bilberry. All potential unigenes involved in flavonoid biosynthetic pathway were determined as DEGs between G and R stages. G and R indicates the two bilberry fruit developmental stages used for construction of transcriptome libraries

**Supplementary Table S6.** DEGs analysis of bilberry ripening-related genes and search against reference sequences with threshold of E-value of 1E-5. All potential unigenes involved in fruit ripening were determined as DEGs between G and R stages. G and R indicates the two bilberry fruit developmental stages used for construction of transcriptome libraries.

**Supplementary Table S7.** Primers used for qRT-PCR analysis.

**Supplementary Figure S1. Size distribution of bilberry unigenes.** Bars represent the number of bilberry unigenes in each given sequence length.

**Supplementary Figure S2. qRT-PCR analysis of anthocyanin biosynthetic genes during bilberry fruit development**. Relative expression of (a) CHS, (b) ANS, and (c) UFGT. Bars represent the relative expression levels of unigenes in each stage normalized with respect to the internal control *GAPDH*. Error bars represent standard error of four biological replicates. CHS, chalcone synthase; ANS, anthocyanidin synthase; UFGT, UDP-glucose flavonoid glucosyltransferase. S1 – Flower, S2 – Small unripe green fruit, S3 – Large unripe green fruit (G), S4 – Ripening purple fruit (R), S5 – Fully ripe blue fruit. G and R indicated two bilberry stages which are used for construction of transcriptome libraries. *Asterisks* indicate significant differences between early stage (S3) and ripening stages (S4, S5) at level p < 0.05^*^, p < 0.01^**^, p < 0.001^***^ using Student’s *t*-Test.

**Supplementary Table S2.** Statistics of Gene Ontology (GO) classification of bilberry transcriptome.

| **GO category** | **GO-Terms** | **Number of unigenes** | **Percent of genes** |
| --- | --- | --- | --- |
| **Biological Process** |  | **23,891** | **66.13 %** |
|  | biological adhesion | 16 | 0.07 |
|  | biological phase | 2 | 0.008 |
|  | biological regulation | 5,321 | 22.27 |
|  | cell killing | 7 | 0.03 |
|  | cellular component organization or biogenesis | 3,781 | 15.83 |
|  | cellular process | 17,497 | 73.24 |
|  | detoxification | 193 | 0.81 |
|  | developmental process | 1,780 | 7.45 |
|  | growth | 328 | 1.37 |
|  | immune system process | 228 | 0.95 |
|  | localization | 4,274 | 17.89 |
|  | locomotion | 23 | 0.10 |
|  | metabolic process | 18,197 | 76.17 |
|  | multicellular organismal process | 1,530 | 6.40 |
|  | multi-organism process | 537 | 2.25 |
|  | negative regulation of biological process | 729 | 3.05 |
|  | positive regulation of biological process | 627 | 2.62 |
|  | regulation of biological process | 4,612 | 19.30 |
|  | reproduction | 886 | 3.71 |
|  | reproductive process | 881 | 3.69 |
|  | response to stimulus | 4,208 | 17.61 |
|  | rhythmic process | 56 | 0.23 |
|  | signaling | 1,544 | 6.46 |
|  | single-organism process | 11,776 | 49.29 |
| **Cellular Component** |  | **23,148** | **64.08 %** |
|  | cell | 17,159 | 74.13 |
|  | cell junction | 616 | 2.66 |
|  | cell part | 17,080 | 73.79 |
|  | extracellular region | 540 | 2.33 |
|  | extracellular region part | 76 | 0.33 |
|  | macromolecular complex | 4,523 | 19.54 |
|  | membrane | 12,689 | 54.82 |
|  | membrane part | 10,231 | 44.20 |
|  | membrane-enclosed lumen | 1,282 | 5.54 |
|  | nucleoid | 41 | 0.18 |
|  | organelle | 12,356 | 53.38 |
|  | organelle part | 6,016 | 25.99 |
|  | supramolecular complex | 148 | 0.64 |
|  | symplast | 615 | 2.66 |
|  | virion | 175 | 0.76 |
|  | virion part | 175 | 0.76 |
| **Molecular Function** |  | **25,937** | **71.80 %** |
|  | antioxidant activity | 184 | 0.71 |
|  | binding | 15,431 | 59.49 |
|  | catalytic activity | 17,160 | 66.16 |
|  | electron carrier activity | 213 | 0.82 |
|  | metallochaperone activity | 4 | 0.02 |
|  | molecular function regulator | 505 | 1.95 |
|  | molecular transducer activity | 203 | 0.78 |
|  | nucleic acid binding transcription factor activity | 523 | 2.02 |
|  | nutrient reservoir activity | 50 | 0.19 |
|  | protein tag | 14 | 0.05 |
|  | signal transducer activity | 361 | 1.39 |
|  | structural molecule activity | 1,106 | 4.26 |
|  | transcription factor activity, protein binding | 198 | 0.76 |
|  | translation regulator activity | 1 | 0.004 |
|  | transporter activity | 2,151 | 8.29 |

**Supplementary Table S3.** Statistics of KEGG orthology (KO) classification of bilberry transcriptome.

| **KEGG orthology (KO)-Hierarchy pathway 1** | **KEGG orthology (KO)-Hierarchy pathway 2** | **KEGG Pathway** | **Pathway ID** | **Number of unigenes** |
| --- | --- | --- | --- | --- |
| **Environmental Information Processing** | **Signal transduction** | Phosphatidylinositol signaling system | map04070 | 140 |
|  |  | mTOR signaling pathway | map04150 | 60 |
| **Genetic Information Processing** | **Translation** | Aminoacyl-tRNA biosynthesis | map00970 | 183 |
| **Metabolism** | **Amino acid metabolism** | Cysteine and methionine metabolism | map00270 | 243 |
|  |  | Glycine, serine and threonine metabolism | map00260 | 224 |
|  |  | Lysine degradation | map00310 | 183 |
|  |  | Valine, leucine and isoleucine degradation | map00280 | 180 |
|  |  | Tryptophan metabolism | map00380 | 172 |
|  |  | Alanine, aspartate and glutamate metabolism | map00250 | 160 |
|  |  | Arginine and proline metabolism | map00330 | 152 |
|  |  | Phenylalanine metabolism | map00360 | 151 |
|  |  | Tyrosine metabolism | map00350 | 141 |
|  |  | Phenylalanine, tyrosine and tryptophan biosynthesis | map00400 | 125 |
|  |  | Histidine metabolism | map00340 | 124 |
|  |  | Arginine biosynthesis | map00220 | 96 |
|  |  | Lysine biosynthesis | map00300 | 47 |
|  |  | Valine, leucine and isoleucine biosynthesis | map00290 | 46 |
|  | **Biosynthesis of antibiotics** | Biosynthesis of antibiotics | map01130 | 1,365 |
|  | **Biosynthesis of other secondary metabolites** | Phenylpropanoid biosynthesis | map00940 | 297 |
|  |  | Flavonoid biosynthesis | map00941 | 105 |
|  |  | Streptomycin biosynthesis | map00521 | 80 |
|  |  | Isoquinoline alkaloid biosynthesis | map00950 | 56 |
|  |  | Tropane, piperidine and pyridine alkaloid biosynthesis | map00960 | 40 |
|  |  | Monobactam biosynthesis | map00261 | 37 |
| **Metabolism** | **Biosynthesis of other secondary metabolites** | Indole alkaloid biosynthesis | map00901 | 37 |
|  |  | Novobiocin biosynthesis | map00401 | 24 |
|  |  | Acarbose and validamycin biosynthesis | map00525 | 22 |
|  |  | Aflatoxin biosynthesis | map00254 | 22 |
|  |  | Flavone and flavonol biosynthesis | map00944 | 21 |
|  |  | Neomycin, kanamycin and gentamicin biosynthesis | map00524 | 21 |
|  |  | Caffeine metabolism | map00232 | 18 |
|  |  | Stilbenoid, diarylheptanoid and gingerol biosynthesis | map00945 | 16 |
|  |  | Glucosinolate biosynthesis | map00966 | 16 |
|  |  | Anthocyanin biosynthesis | map00942 | 13 |
|  |  | Betalain biosynthesis | map00965 | 5 |
|  |  | Carbapenem biosynthesis | map00332 | 4 |
|  |  | Penicillin and cephalosporin biosynthesis | map00311 | 2 |
|  |  | Isoflavonoid biosynthesis | map00943 | 2 |
|  |  | Benzoxazinoid biosynthesis | map00402 | 1 |
|  | **Biosynthesis of terpenoids and steroids** | Biosynthesis of terpenoids and steroids | map01062 | 5 |
|  | **Carbohydrate metabolism** | Starch and sucrose metabolism | map00500 | 578 |
|  |  | Amino sugar and nucleotide sugar metabolism | map00520 | 389 |
|  |  | Glycolysis / Gluconeogenesis | map00010 | 383 |
|  |  | Pyruvate metabolism | map00620 | 374 |
|  |  | Galactose metabolism | map00052 | 285 |
|  |  | Glyoxylate and dicarboxylate metabolism | map00630 | 240 |
|  |  | Pentose and glucuronate interconversions | map00040 | 232 |
|  |  | Citrate cycle (TCA cycle) | map00020 | 220 |
|  |  | Pentose phosphate pathway | map00030 | 202 |
|  |  | Fructose and mannose metabolism | map00051 | 187 |
|  |  | Ascorbate and aldarate metabolism | map00053 | 185 |
| **Metabolism** | **Carbohydrate metabolism** | Inositol phosphate metabolism | map00562 | 160 |
|  |  | Propanoate metabolism | map00640 | 130 |
|  |  | Butanoate metabolism | map00650 | 94 |
|  |  | C5-Branched dibasic acid metabolism | map00660 | 36 |
|  | **Energy metabolism** | Oxidative phosphorylation | map00190 | 244 |
|  |  | Methane metabolism | map00680 | 244 |
|  |  | Carbon fixation in photosynthetic organisms | map00710 | 235 |
|  |  | Carbon fixation pathways in prokaryotes | map00720 | 211 |
|  |  | Sulfur metabolism | map00920 | 81 |
|  |  | Nitrogen metabolism | map00910 | 75 |
|  |  | Photosynthesis | map00195 | 20 |
|  | **Glycan biosynthesis and metabolism** | Other glycan degradation | map00511 | 172 |
|  |  | Glycosaminoglycan degradation | map00531 | 82 |
|  |  | Glycosphingolipid biosynthesis - ganglio series | map00604 | 79 |
|  |  | N-Glycan biosynthesis | map00510 | 61 |
|  |  | Various types of N-glycan biosynthesis | map00513 | 43 |
|  |  | Glycosaminoglycan biosynthesis - heparan sulfate / heparin | map00534 | 25 |
|  |  | Glycosphingolipid biosynthesis - globo and isoglobo series | map00603 | 23 |
|  |  | Other types of O-glycan biosynthesis | map00514 | 20 |
|  |  | Glycosaminoglycan biosynthesis - chondroitin sulfate / dermatan sulfate | map00532 | 17 |
|  |  | Lipopolysaccharide biosynthesis | map00540 | 16 |
|  |  | Peptidoglycan biosynthesis | map00550 | 6 |
|  |  | Glycosphingolipid biosynthesis - lacto and neolacto series | map00601 | 4 |
|  |  | Glycosylphosphatidylinositol (GPI)-anchor biosynthesis | map00563 | 2 |
|  |  | Glycosaminoglycan biosynthesis - keratan sulfate | map00533 | 1 |
|  |  | Mucin type O-glycan biosynthesis | map00512 | 1 |
| **Metabolism** | **Lipid metabolism** | Fatty acid degradation | map00071 | 203 |
|  |  | Glycerolipid metabolism | map00561 | 202 |
|  |  | Glycerophospholipid metabolism | map00564 | 161 |
|  |  | Sphingolipid metabolism | map00600 | 152 |
|  |  | Fatty acid biosynthesis | map00061 | 135 |
|  |  | alpha-Linolenic acid metabolism | map00592 | 115 |
|  |  | Biosynthesis of unsaturated fatty acids | map01040 | 93 |
|  |  | Steroid hormone biosynthesis | map00140 | 81 |
|  |  | Arachidonic acid metabolism | map00590 | 63 |
|  |  | Steroid biosynthesis | map00100 | 61 |
|  |  | Ether lipid metabolism | map00565 | 59 |
|  |  | Fatty acid elongation | map00062 | 53 |
|  |  | Linoleic acid metabolism | map00591 | 36 |
|  |  | Cutin, suberine and wax biosynthesis | map00073 | 34 |
|  |  | Primary bile acid biosynthesis | map00120 | 27 |
|  |  | Synthesis and degradation of ketone bodies | map00072 | 15 |
|  | **Metabolism of cofactors and vitamins** | Thiamine metabolism | map00730 | 1,629 |
|  |  | Porphyrin and chlorophyll metabolism | map00860 | 141 |
|  |  | Nicotinate and nicotinamide metabolism | map00760 | 96 |
|  |  | One carbon pool by folate | map00670 | 82 |
|  |  | Pantothenate and CoA biosynthesis | map00770 | 70 |
|  |  | Retinol metabolism | map00830 | 68 |
|  |  | Biotin metabolism | map00780 | 60 |
|  |  | Ubiquinone and other terpenoid-quinone biosynthesis | map00130 | 58 |
|  |  | Vitamin B6 metabolism | map00750 | 39 |
|  |  | Folate biosynthesis | map00790 | 34 |
|  |  | Riboflavin metabolism | map00740 | 15 |
|  |  | Lipoic acid metabolism | map00785 | 6 |
| **Metabolism** | **Metabolism of other amino acids** | Glutathione metabolism | map00480 | 230 |
|  |  | beta-Alanine metabolism | map00410 | 163 |
|  |  | Cyanoamino acid metabolism | map00460 | 152 |
|  |  | Selenocompound metabolism | map00450 | 67 |
|  |  | D-Glutamine and D-glutamate metabolism | map00471 | 18 |
|  |  | Phosphonate and phosphinate metabolism | map00440 | 10 |
|  |  | Taurine and hypotaurine metabolism | map00430 | 10 |
|  |  | D-Arginine and D-ornithine metabolism | map00472 | 6 |
|  |  | D-Alanine metabolism | map00473 | 2 |
|  | **Metabolism of terpenoids and polyketides** | Terpenoid backbone biosynthesis | map00900 | 116 |
|  |  | Limonene and pinene degradation | map00903 | 75 |
|  |  | Carotenoid biosynthesis | map00906 | 49 |
|  |  | Geraniol degradation | map00281 | 39 |
|  |  | Polyketide sugar unit biosynthesis | map00523 | 25 |
|  |  | Biosynthesis of vancomycin group antibiotics | map01055 | 22 |
|  |  | Tetracycline biosynthesis | map00253 | 22 |
|  |  | Diterpenoid biosynthesis | map00904 | 17 |
|  |  | Biosynthesis of ansamycins | map01051 | 15 |
|  |  | Sesquiterpenoid and triterpenoid biosynthesis | map00909 | 10 |
|  |  | Zeatin biosynthesis | map00908 | 5 |
|  |  | Monoterpenoid biosynthesis | map00902 | 4 |
|  | **Metabolism of terpenoids and polyketides** | Biosynthesis of siderophore group nonribosomal peptides | map01053 | 3 |
|  |  | Insect hormone biosynthesis | map00981 | 2 |
|  | **Nucleotide metabolism** | Purine metabolism | map00230 | 2,052 |
|  |  | Pyrimidine metabolism | map00240 | 361 |
|  | **Xenobiotics biodegradation and metabolism** | Aminobenzoate degradation | map00627 | 685 |
|  |  | Drug metabolism - other enzymes | map00983 | 321 |
| **Metabolism** | **Xenobiotics biodegradation and metabolism** | Drug metabolism - cytochrome P450 | map00982 | 208 |
|  |  | Metabolism of xenobiotics by cytochrome P450 | map00980 | 191 |
|  |  | Chloroalkane and chloroalkene degradation | map00625 | 85 |
|  |  | Benzoate degradation | map00362 | 50 |
|  |  | Steroid degradation | map00984 | 36 |
|  |  | Toluene degradation | map00623 | 31 |
|  |  | Caprolactam degradation | map00930 | 28 |
|  |  | Styrene degradation | map00643 | 23 |
|  |  | Naphthalene degradation | map00626 | 21 |
|  |  | Ethylbenzene degradation | map00642 | 16 |
|  |  | Fluorobenzoate degradation | map00364 | 15 |
|  |  | Chlorocyclohexane and chlorobenzene degradation | map00361 | 15 |
|  |  | Atrazine degradation | map00791 | 5 |
|  |  | Xylene degradation | map00622 | 2 |
|  |  | Polycyclic aromatic hydrocarbon degradation | map00624 | 1 |
|  |  | Dioxin degradation | map00621 | 1 |
| **Organismal Systems** | **Immune system** | T cell receptor signaling pathway | map04660 | 416 |

**Supplementary Table S4.** GO enrichment analysis of bilberry DEGs. The numbers of up- and downregulated DEGs in R stage compared to G stage which were assigned into 46 over-represented GO terms (p-value < 0.05) of three main categories: BP, Biological process; CC, Cellular component; MF, Molecular function.

| **DEGs** | **GO-ID** | **Term** | **Category** | **FDR** | **P-Value** | **Unigenes** |
| --- | --- | --- | --- | --- | --- | --- |
| **Upreguated** | GO:0009813 | flavonoid biosynthetic process | BP | 5.04E-11 | 2.48E-14 | 39 |
| **Upreguated** | GO:0045490 | pectin catabolic process | BP | 5.21E-06 | 1.30E-08 | 16 |
| **Upreguated** | GO:0052696 | flavonoid glucuronidation | BP | 1.23E-05 | 4.28E-08 | 25 |
| **Upreguated** | GO:0009800 | cinnamic acid biosynthetic process | BP | 1.05E-04 | 5.65E-07 | 7 |
| **Upreguated** | GO:0006559 | L-phenylalanine catabolic process | BP | 1.47E-03 | 1.30E-05 | 7 |
| **Upreguated** | GO:0009638 | phototropism | BP | 1.98E-03 | 2.01E-05 | 6 |
| **Upreguated** | GO:0006679 | glucosylceramide biosynthetic process | BP | 4.05E-03 | 4.95E-05 | 6 |
| **Upreguated** | GO:0055114 | oxidation-reduction process | BP | 2.14E-02 | 3.69E-04 | 184 |
| **Upreguated** | GO:0010117 | photoprotection | BP | 2.58E-02 | 4.61E-04 | 4 |
| **Upreguated** | GO:0006022 | aminoglycan metabolic process | BP | 2.59E-02 | 4.66E-04 | 9 |
| **Upreguated** | GO:0006749 | glutathione metabolic process | BP | 3.53E-02 | 7.17E-04 | 13 |
| **Upreguated** | GO:0009664 | plant-type cell wall organization | BP | 3.56E-02 | 7.47E-04 | 12 |
| **Upreguated** | GO:0098661 | inorganic anion transmembrane transport | BP | 3.58E-02 | 7.59E-04 | 7 |
| **Upreguated** | GO:0044036 | cell wall macromolecule metabolic process | BP | 3.66E-02 | 7.88E-04 | 17 |
| **Upreguated** | GO:0005576 | extracellular region | CC | 7.70E-04 | 6.05E-06 | 49 |
| **Upreguated** | GO:0005618 | cell wall | CC | 1.73E-03 | 1.60E-05 | 46 |
| **Upreguated** | GO:0016210 | naringenin-chalcone synthase activity | MF | 4.03E-06 | 8.04E-09 | 8 |
| **Upreguated** | GO:0030570 | pectate lyase activity | MF | 5.06E-06 | 1.20E-08 | 11 |
| **Upreguated** | GO:0080043 | quercetin 3-O-glucosyltransferase activity | MF | 1.13E-05 | 3.37E-08 | 23 |
| **Upreguated** | GO:0080044 | quercetin 7-O-glucosyltransferase activity | MF | 1.13E-05 | 3.37E-08 | 23 |
| **Upreguated** | GO:0045548 | phenylalanine ammonia-lyase activity | MF | 2.31E-05 | 9.24E-08 | 8 |
| **Upreguated** | GO:0033772 | flavonoid 3',5'-hydroxylase activity | MF | 2.07E-03 | 2.17E-05 | 5 |
| **Upreguated** | GO:0008120 | ceramide glucosyltransferase activity | MF | 4.05E-03 | 4.95E-05 | 6 |
| **Upreguated** | GO:0008061 | chitin binding | MF | 7.67E-03 | 1.06E-04 | 6 |
| **Upreguated** | GO:0016405 | CoA-ligase activity | MF | 1.17E-02 | 1.77E-04 | 10 |
| **Upreguated** | GO:0004322 | ferroxidase activity | MF | 1.23E-02 | 1.90E-04 | 5 |
| **Upreguated** | GO:0008199 | ferric iron binding | MF | 1.23E-02 | 1.90E-04 | 5 |
| **Upreguated** | GO:0016878 | acid-thiol ligase activity | MF | 2.14E-02 | 3.70E-04 | 10 |
| **Upreguated** | GO:0080124 | pheophytinase activity | MF | 2.14E-02 | 3.72E-04 | 3 |
| **Upreguated** | GO:0016711 | flavonoid 3'-monooxygenase activity | MF | 2.58E-02 | 4.61E-04 | 4 |
| **Upreguated** | GO:0008113 | peptide-methionine (S)-S-oxide reductase activity | MF | 3.56E-02 | 7.40E-04 | 4 |
| **Upreguated** | GO:0016710 | trans-cinnamate 4-monooxygenase activity | MF | 4.02E-02 | 8.97E-04 | 3 |
| **Upreguated** | GO:0015415 | ATPase-coupled phosphate ion transmembrane transporter activity | MF | 4.02E-02 | 8.97E-04 | 3 |
| **Upreguated** | GO:0008810 | cellulase activity | MF | 4.20E-02 | 9.59E-04 | 6 |
| **Upreguated** | GO:0046912 | transferase activity, transferring acyl groups, acyl groups converted into alkyl on transfer | MF | 4.60E-02 | 1.05E-03 | 8 |
| **Upreguated** | GO:0004364 | glutathione transferase activity | MF | 4.75E-02 | 1.11E-03 | 12 |
| **Downregulated** | GO:0009768 | photosynthesis, light harvesting in photosystem I | BP | 4.01E-04 | 1.40E-06 | 7 |
| **Downregulated** | GO:0018298 | protein-chromophore linkage | BP | 6.13E-03 | 2.45E-05 | 7 |
| **Downregulated** | GO:0009535 | chloroplast thylakoid membrane | CC | 8.15E-06 | 1.19E-08 | 18 |
| **Downregulated** | GO:0009522 | photosystem I | CC | 3.89E-04 | 1.31E-06 | 9 |
| **Downregulated** | GO:0009654 | photosystem II oxygen evolving complex | CC | 1.87E-03 | 7.00E-06 | 5 |
| **Downregulated** | GO:0045735 | nutrient reservoir activity | MF | 2.15E-14 | 2.69E-18 | 15 |
| **Downregulated** | GO:0016168 | chlorophyll binding | MF | 9.46E-06 | 1.65E-08 | 10 |
| **Downregulated** | GO:0031409 | pigment binding | MF | 4.65E-03 | 1.80E-05 | 6 |
| **Downregulated** | GO:0016709 | oxidoreductase activity, acting on paired donors, with incorporation or reduction of molecular oxygen, NAD(P)H as one donor, and incorporation of one atom of oxygen | MF | 1.14E-02 | 5.56E-05 | 10 |
| **Downregulated** | GO:0008465 | glycerate dehydrogenase activity | MF | 2.18E-02 | 1.11E-04 | 2 |

BP, Biological Process; CC, Cellular Component; MF, Molecular Function

**Supplementary Table S5.** DEGs analysis of flavonoid genes in bilberry. All potential unigenes involved in flavonoid biosynthetic pathway were determined as DEGs between G and R stages. G and R indicates the two bilberry fruit developmental stages used for construction of transcriptome libraries

| Gene_name | Gene_id | Description | G (TPM) ^a^ | R (TPM) ^a^ | Fold-change ^a^ | Regulation |
| --- | --- | --- | --- | --- | --- | --- |
| PAL | c12892_g3_i2 ^a^ | Phenylalanine ammonia-lyase | 3.06 | 41.02 | 3.74 | UP |
|  | c11579_g2_i2 ^a^ | Phenylalanine ammonia- partial | 3.17 | 26.75 | 3.08 | UP |
|  | c26371_g1_i1 ^a^ | Phenylalanine ammonia- partial | 2.92 | 20.58 | 2.82 | UP |
|  | c12648_g5_i1 ^a^ | Phenylalanine ammonia-lyase | 3.16 | 20.04 | 2.66 | UP |
|  | c12648_g3_i1 ^a,b,c^ | Phenylalanine ammonia-lyase | 4.10 | 105.11 | 4.68 | UP |
| C4H | c12763_g1_i1 ^a^ | Trans-cinnamate 4-monooxygenase-like | 2.22 | 116.78 | 5.72 | UP |
| 4CL | c11857_g1_i2 ^a^ | 4-coumarate-- ligase-like 7 | 3.41 | 27.60 | 3.02 | UP |
|  | c12099_g7_i1 ^a^ | 4-coumarate-- ligase 2 | 1.20 | 30.15 | 4.65 | UP |
|  | c10332_g2_i1 ^a,b,c^ | 4-coumarate- partial | 0.00 | 23.66 | +inf | UP |
|  | c10332_g1_i1 ^a,b,c^ | 4-coumarate-- ligase 2 | 0.00 | 22.54 | +inf | UP |
|  | c10153_g1_i2 ^a^ | 4-coumarate-- ligase-like 5 | 32.11 | 0.00 | -inf | DOWN |
| CHS | c18177_g1_i1 ^a^ | Chalcone synthase | 0.00 | 20.27 | +inf | UP |
|  | c12440_g3_i3 ^a^ | Chalcone synthase | 0.00 | 40.28 | +inf | UP |
|  | c12904_g2_i1 ^a,b,c^ | Chalcone partial | 45.29 | 246.13 | 2.44 | UP |
|  | c12657_g2_i1 ^a^ | Chalcone synthase | 13.01 | 130.18 | 3.32 | UP |
|  | c12301_g2_i1 ^a^ | Chalcone synthase | 4.01 | 151.36 | 5.24 | UP |
|  | c12755_g3_i2 ^a,b,c^ | Chalcone synthase | 33.68 | 709.40 | 4.40 | UP |
| CHI | c11762_g2_i2 ^a,b,c^ | Chalcone isomerase | 9.84 | 234.93 | 4.58 | UP |
|  | c12896_g1_i7 ^a^ | probable Chalcone--flavonone isomerase 3 | 8.55 | 59.48 | 2.80 | UP |
| F3H | c12890_g3_i1 ^a,b,c^ | Flavanone 3-hydroxylase | 1.83 | 193.72 | 6.73 | UP |
|  | c12900_g3_i2 ^a^ | Flavonol synthase flavanone 3-hydroxylase-like | 0.00 | 24.60 | +inf | UP |
|  | c11315_g7_i1 ^a,b,c^ | Naringenin,2-oxoglutarate 3-dioxygenase | 9.15 | 160.34 | 4.13 | UP |
| F3'H | c11619_g5_i1 ^a^ | Flavonoid 3 -monooxygenase-like | 2.93 | 22.70 | 2.95 | UP |
|  | c11184_g4_i5 ^a^ | Flavonoid 3 -hydroxylase | 0.00 | 194.81 | +inf | UP |
| F3'5'H  F3’5’H | c8625_g1_i1 ^a,b,c^ | Flavonoid 3 ,5 -hydroxylase 2-like | 0.00 | 27.34 | +inf | UP |
|  | c12783_g3_i5 ^a^ | Flavonoid 3 ,5 -hydroxylase 2-like | 0.45 | 27.77 | 5.95 | UP |
|  | c12783_g3_i3 ^a,b,c^ | Flavonoid 3 ,5 -hydroxylase 2-like | 3.90 | 151.67 | 5.28 | UP |
|  | c13557_g1_i1 ^a^ | Flavonoid 3 ,5 -hydroxylase 2-like | 3.55 | 21.54 | 2.60 | UP |
|  | c9198_g1_i1 ^a^ | Flavonoid 3 ,5 -hydroxylase | 2.73 | 20.38 | 2.90 | UP |
|  | c11888_g3_i1 ^a,b,c^ | Flavonoid 3 ,5 -hydroxylase | 87.60 | 1.50 | -5.87 | DOWN |
| ANS | c12683_g5_i4 ^a^ | Leucoanthocyanidin dioxygenase | 4.57 | 1,595.04 | 8.45 | UP |
|  | c11284_g1_i1 ^a,b,c^ | Anthocyanidin synthase | 21.19 | 616.96 | 4.86 | UP |
|  | c9455_g1_i1 ^a^ | Leucoanthocyanidin dioxygenase | 0.00 | 22.87 | +inf | UP |
| UFGT | c12490_g1_i1 ^a^ | Kaempferol 3-O-beta-D-galactosyltransferase-like | 0.37 | 305.67 | 9.69 | UP |
| OMT | c12654_g1_i1 ^a,b,c^ | probable Caffeoyl- O-methyltransferase | 8.61 | 1,690.42 | 7.62 | UP |
|  | c13264_g1_i1 ^a^ | Caffeic acid O-methyltransferase | 3.27 | 20.07 | 2.62 | UP |
|  | c12808_g3_i1 ^a,b,c^ | Caffeoyl- O-methyltransferase | 14.96 | 127.97 | 3.10 | UP |
| LAR | c8871_g1_i1 ^a,b,c^ | Leucoanthocyanidin reductase | 6.21 | 55.60 | 3.16 | UP |
| PPO | c10796_g1_i3 ^a,b,c^ | Polyphenol oxidase | 0.93 | 36.44 | 5.29 | UP |
| FST | c4605_g1_i1 ^a^ | Flavonol sulfotransferase-like | 2.42 | 20.57 | 3.09 | UP |
|  | c11428_g1_i1 ^a^ | Flavonol sulfotransferase-like | 1.61 | 26.31 | 4.03 | UP |
|  | c12720_g2_i4 ^a^ | Flavonol sulfotransferase-like | 22.83 | 258.19 | 3.50 | UP |
|  | c12720_g10_i1 ^a^ | Flavonol sulfotransferase-like | 5.02 | 80.50 | 4.00 | UP |
| GST | c12035_g1_i3 ^a,b,c^ | Glutathione S-transferase F11-like | 1.20 | 41.32 | 5.11 | UP |
|  | c9947_g1_i1 ^a,b,c^ | Glutathione S-transferase | 1.50 | 26.96 | 4.17 | UP |
|  | c12738_g3_i2 ^a,b,c^ | Glutathione S-transferase-like | 0.00 | 33.21 | +inf | UP |
|  | c12035_g1_i2 ^a^ | Glutathione S-transferase | 0.00 | 674.30 | +inf | UP |
|  | c11186_g2_i1 ^a^ | Glutathione transferase GST 23-like | 2.02 | 32.65 | 4.01 | UP |
|  | c11941_g1_i2 ^a^ | Glutathione S-transferase zeta class-like | 2.58 | 38.39 | 3.90 | UP |
|  | c7178_g1_i3 ^a^ | Glutathione S-transferase T1-like | 25.90 | 4.92 | -2.40 | DOWN |
|  | c11277_g3_i2 ^a^ | Glutathione S-transferase T1-like | 0.00 | 22.06 | +inf | UP |
|  | c11769_g2_i2 ^a^ | Glutathione S-transferase T1-like | 12.65 | 84.09 | 2.73 | UP |
|  | c19820_g2_i1 ^a^ | probable glutathione S-transferase | 0.00 | 20.38 | +inf | UP |
|  | c5375_g1_i1 ^a^ | Glutathione S-transferase 2 | 4.24 | 22.61 | 2.41 | UP |

PAL, Phenylalanine ammonia lyase; C4H, Cinnamate 4-hydroxylase; 4CL, 4 coumarate CoA ligase; CHS, Chalcone synthase; CHI, Chalcone isomerase; F3H, Flavanone 3-hydroxylase; F3’H, Flavonoid 3’-hydroxylase; F3’5’H, Flavonoids 3’,5’-hydroxylase; FLS, Flavonol synthase; DFR, Dihydroflavonol 4-reductase; ANS, Anthocyanin synthase; UFGT, UDP-glucose flavonoid glucosyltransferase; OMT, O-methyltransferase; LAR, Leucoanthocyanidin reductase; PPO, Polyphenol oxidase, FST, Flavonol 3-sulfotransferase; GST, Glutathione S-transferase.

a indicates the unigenes that are determined as DEGs by Kallisto method, significantly different expression was considered with absolute value of log fold change > 2.3 and threshold of TMP value >20.

b indicates indicate the unigenes that are determined as DEGs by DESeq2 method, significantly different expression was considered with absolute value of log fold change > 2 and p-value < 0.1.

c indicates indicate the unigenes that are determined as DEGs by EdgeR method, significantly different expression was considered with absolute value of log fold change > 2 and p-value < 0.01.

+/- inf = +/- infinity

**Supplementary Table S6.** DEGs analysis of bilberry ripening-related genes and search against reference sequences with threshold of E-value of 1E-5. All potential unigenes involved in fruit ripening were determined as DEGs between G and R stages. G and R indicates the two bilberry fruit developmental stages used for construction of transcriptome libraries.

| Gene_name | Gene_id | Description | G (TPM)^a^ | R (TPM)^a^ | Fold change^a^ | Regulation | Ref-seq | Protein_id | % identity | E_value |
| --- | --- | --- | --- | --- | --- | --- | --- | --- | --- | --- |
| MADS | c11556_g3_i3^a^ | MADS-domain transcription partial | 0.00 | 25.93 | +inf | UP |  |  |  |  |
|  | c11823_g2_i3 ^a^ | Floral-binding partial | 0.73 | 23.47 | 5.01 | UP | *SlMADS1^72^* | AAP57412.1 | 65.022 | 2.95E-92 |
|  | c12746_g6_i2 ^a^ | Truncated transcription factor CAULIFLOWER A-like isoform X1 | 19.50 | 489.70 | 4.65 | UP | *VmTDR4^4^* | ACR19996.1 | 100 | 4.61E-65 |
|  | c10873_g3_i1 ^a^ | AGAMOUS-like 66 isoform 1 | 4.19 | 44.76 | 3.42 | UP |  |  |  |  |
|  | c7167_g1_i1 ^a^ | SQUAMOSA promoter-binding -like (SBP domain) transcription factor family partial | 2.94 | 23.35 | 2.99 | UP | *CsSPL6* | AOO19736.1 | 63 | 3E-10 |
| NAC | c22060_g1_i1 ^a^ | NAC domain-containing 72-like | 0.00 | 30.28 | +inf | UP |  |  |  |  |
|  | c153_g1_i1 ^a^ | NAC domain-containing 89-like | 0.00 | 21.97 | +inf | UP |  |  |  |  |
|  | c22491_g1_i1 ^a^ | NAC domain-containing 17-like | 0.00 | 20.67 | +inf | UP | *PpNAC^43^* | XP_007210663.1 | 63.953 | 2.76E-31 |
|  | c12000_g2_i5 ^a^ | NAC domain-containing 72-like | 6.05 | 45.14 | 2.90 | UP | *LeNOR^19^* | AAU43922.1 | 66.471 | 4.01E-74 |
|  | c11625_g4_i3 ^a^ | NAC transcription factor 29-like | 0.00 | 112.10 | +inf | UP | *LeNOR^19^* | AAU43922.1 | 67.059 | 3.07E-78 |
|  | c3865_g1_i1 ^a,b,c^ | No apical meristem family | 23.80 | 0.00 | -inf | DOWN |  |  |  |  |
| WRKY | c9726_g1_i1 ^a^ | probable WRKY transcription factor 23 | 2.85 | 24.70 | 3.12 | UP |  |  |  |  |
|  | c735_g1_i1 ^a^ | probable WRKY transcription factor 32 | 2.57 | 27.42 | 3.42 | UP |  |  |  |  |
|  | c12156_g3_i1^c^ | probable WRKY transcription factor 40 | 0.00 | 23.21 | +inf | UP |  |  |  |  |
|  | c10987_g4_i1 ^a,b,c^ | probable WRKY transcription factor 40 | 3.56 | 54.94 | 3.95 | UP |  |  |  |  |
| SPATULA | c2041_g1_i1 ^a^ | Transcription factor SPATULA-like | 0.00 | 20.67 | +inf | UP | *VcHLH032* | AOY34403.1 | 100 | 4E-55 |
| LOB  LOB | c10885_g3_i5 ^a^ | LOB domain-containing 38-like | 41.01 | 1.95 | -4.39 | DOWN | *AtLBD39^44^* | NP_195470.1 | 67.939 | 1.96E-59 |
|  | c8553_g1_i1 ^a,b^ | LOB domain-containing 20 | 32.81 | 0.00 | -inf | DOWN | *VvLBD^45^* | XP_002279521.1 | 77.632 | 2.69E-33 |
|  | c11398_g8_i1 ^b,c^ | LOB domain-containing 25 | 19.00 ^b,c^ | 0.00 ^b,c^ | -7.69 ^b^ | DOWN |  |  |  |  |
|  | c11341_g1_i4 ^b^ | LOB domain-containing 41 | 11.00 ^b^ | 0.00 ^b^ | -6.9 ^b^ | DOWN |  |  |  |  |
| AP2/ERF | c11109_g8_i1 ^a^ | Ethylene-responsive transcription factor RAP2-4-like | 3.97 | 20.87 | 2.39 | UP | *LeEFR1^52^* | AAL75809.1 | 75 | 8.79E-12 |
|  | c10506_g1_i2 ^a^ | Ethylene-responsive transcription factor RAP2-4-like | 10.81 | 56.33 | 2.38 | UP |  |  |  |  |
| ERF | c26745_g1_i1 ^a^ | Ethylene-responsive transcription factor ERF014 | 0.00 | 26.27 | +inf | UP |  |  |  |  |
|  | c12669_g1_i1 ^a^ | Ethylene-responsive transcription factor ERF003-like | 0.00 | 46.96 | +inf | UP | *LeEFR1^52^* | AAL75809.1 | 51.63 | 2.58E-28 |
|  | c10464_g1_i3 ^a,b,c^ | Ethylene-responsive transcription factor ERF017-like | 25.95 | 2.40 | -3.43 | DOWN | *LeEFR1^52^* | AAL75809.1 | 57.576 | 2.51E-07 |
|  | c11910_g1_i2 ^a,b,c^ | Ethylene-responsive transcription factor ERF062-like | 0.88 | 22.51 | 4.68 | UP | *LeEFR1^52^* | AAL75809.1 | 63.158 | 5.42E-13 |
|  | c11918_g3_i4 ^a^ | Ethylene-responsive transcription factor ERF061 | 0.31 | 34.14 | 6.78 | UP |  |  |  |  |
|  | c9006_g1_i1 ^a^ | Ethylene-responsive transcription factor 3-like | 48.38 | 7.23 | -2.74 | DOWN | *LeERF2^73^* | AAP32202.1 | 80.645 | 3.48E-20 |
| EIL | c11689_g1_i5 ^a^ | ETHYLENE INSENSITIVE 3-like | 0.00 | 27.74 | +inf | UP | *SlEIL2^73^* | AAK58858.1 | 51 | 1.54E-22 |
| ARF | c9212_g1_i2 ^a,b,c^ | Auxin response factor 2-like | 45.24 | 1.50 | -4.91 | DOWN | *SlARF4^56^* | NP_001233771.1 | 45.652 | 2.91E-23 |
|  | c26081_g1_i1 ^a^ | Auxin response factor 19-like | 0.00 | 27.97 | +inf | UP | *SlARF4^56^* | NP_001233771.1 | 37.097 | 4.70E-09 |
| ABI | c5971_g1_i1 ^a^ | ABSCISIC ACID-INSENSITIVE 5 | 1.85 | 23.83 | 3.69 | UP | *VvABF2^58^* | Q9M4H1 | 53.763 | 1.30E-09 |
|  | c12099_g3_i1 ^a,b,c^ | ABSCISIC ACID-INSENSITIVE 5 5 | 1.52 | 78.33 | 5.69 | UP | *VvABF2^58^* | Q9M4H1 | 57.787 | 1.38E-59 |

a indicates the unigenes that are determined as DEGs by Kallisto method, significantly different expression was considered with absolute value of log fold change > 2.3 and threshold of TMP value >20.

b indicates indicate the unigenes that are determined as DEGs by DESeq2 method, significantly different expression was considered with absolute value of log fold change > 2 and p-value < 0.1.

c indicates indicate the unigenes that are determined as DEGs by EdgeR method, significantly different expression was considered with absolute value of log fold change > 2 and p-value < 0.01.

+/- inf = +/- infinity

**Supplementary Table S7.** Primers used for qRT-PCR analysis.

| **Gene** | **Forward primer sequence** | **Reverse primer sequence** |
| --- | --- | --- |
| **GAPDH** | CAA ACT GTC TTG CCC CAC TT | CAG GCA ACA CCT TAC CAA CA |
| **CHS** | CCA AGG CCA TCA AGG AAT G | TGA TAC ATC ATG AGT CGC TTC AC |
| **ANS** | GCA ACT CTT CTA CGA GGG CAA A | CCT GTG GAG AAT GCT CTT GCA C |
| **UFGT** | CAT CCA AAC CCT GTT CCC ATC C | TCA TCC CTG CCT TCA AGC TCT C |
| **MADS**(c11556_g3_i3) | GAA GAT CTG CTT GCT CTT GTG G | TTA TCG ACA AGC CCG TAC TGT G |
| **MADS**(c11823_g2_i3) | GTT CTC TGT GAT GCT GAG GTT | TCC AGT GAT CCA TAG CTA CAC C |
| **MADS**(c10873_g3_i1) | AGC CAG CCT GAC AAA CTG AAA G | TCT CAC GTT GCT GCC TAT TGA G |
| **MADS**(c7167_g1_i1) | CAG GGA GCG TCT TTC TTG TGA A | GTG AGC CGA AGG CAT CAT TTT C |
| **NAC**(c22060_g1_i1) | TGT TTG CTA GGT CTT GCT CTC G | CTC CAT CGT GCT GAA TGC CTA T |
| **NAC**(c153_g1_i1) | CGG CCT TAA TGA CCG AGA ATG | CAG GCT GAG AAA GCT CGT TTG |
| **NAC**(c22491_g1_i1) | GGA GTT GCC TGG TCT CTC TAA A | ATA CCC TTG CCT AGT TGC CCT A |
| **NAC**(c12000_g2_i5) | GTC GGA ATC AAG AAA GCT CTG G | ACA CAG AAC CCA ATC GTC CAA C |
| **NAC**(c11625_g4_i3) | AAG CAT GGG AAG AAC AGA GGA G | CCA CAG GTG AGA AAG TGA GCA A |
| **WRKY**(c9726_g1_i1) | GCT GCA CCA GTG CTA GTT GTA A | TAT GAG GTA GGA CGA GGC ATG A |
| **WRKY**(c12156_g3_i1) | GGC AGG ACG GTG ATC AAT ACT | CTC ATG CTG CTG GAC CAT TCT |
| **WRKY**(c10987_g4_i1) | CAA CCC TTG TCC AAG AGC TTA C | CTT CAG TTC TTG ATG GGT CTG G |
| **SPATULA**(c2041_g1_i1) | GGA TGG AAT TAC GGC GAT GTT C | GCG GTT GAG CTG GAT TTA TTG G |
| **LOB**(c10885_g3_i5) | GGT TCT CTC CGA AGG AAC TTT G | CCG TCA TCG AAT TCT TCT CGA C |
| **AP2/ERF**(c11109_g8_i1) | AAT TGG GCT CAA CCA CCT TAC C | ACA GGG TTT GGG TTT GGA GTG A |
| **AP2/ERF**(c10506_g1_i2) | CAT TCC TCA GTT GAC GCG AAG | GAT CTC GTC CGA TCC TCG AAA |
| **ERF**(c26745_g1_i1) | ACC ATC CCA AGG TAC CTA CAA | TCG TCG TAG ACA TCT TCC ATC |
| **ERF**(c10464_g1_i3) | AAT GGG AAA TCA GGC GAG AG | GGC AGT TGG GTA GTC GGA TCT |
| **ERF**(c11910_g1_i2) | GAA CAG AAC AAG GGT GTG GCT A | CTG AGT TGG CCT TGA GTT GAT G |
| **ERF**(c11918_g3_i4) | GAG TGC ATT GAA GAG TGC AGT G | GCT GTC ATT ACC AAC AGC CTT C |
| **ERF**(c9006_g1_i1) | CAA CGG ATC TGG ATC GAT TAG G | CCA AAC CCT AGC CTT CTT CCA G |
| **EIL**(c11689_g1_i5) | AAG ATC AGA ACG TGT CGC ACT C | GAT TCA ACA CCT TGC ACT GGT C |
| **ARF**(c9212_g1_i2) | AAC TGT TCT CCC AGT GCT AAC C | CAC GGA TGA GTT CTC CAT AAC C |
| **ARF**(c26081_g1_i1) | GAC AGC TGG AGG ACC GAC ATA G | CCG GAC ACA ATT TAC GAA CTC C |
| **ABI**(c5971_g1_i1) | AGC TGG AGA GAA AGA GGA AGC A | AAC TGA AAG TCC TCC GCA TCA C |
| **ABI**(c12099_g3_i1) | GCT GGT GTT GTT AGG GAA GAC A | CCC AAA TCC CGA AGC ACT ACT A |

F, Forward; R, Reverse

**Supplementary Figure S1. Size distribution of bilberry unigenes.** Bars represent the number of bilberry unigenes in each given sequence length.


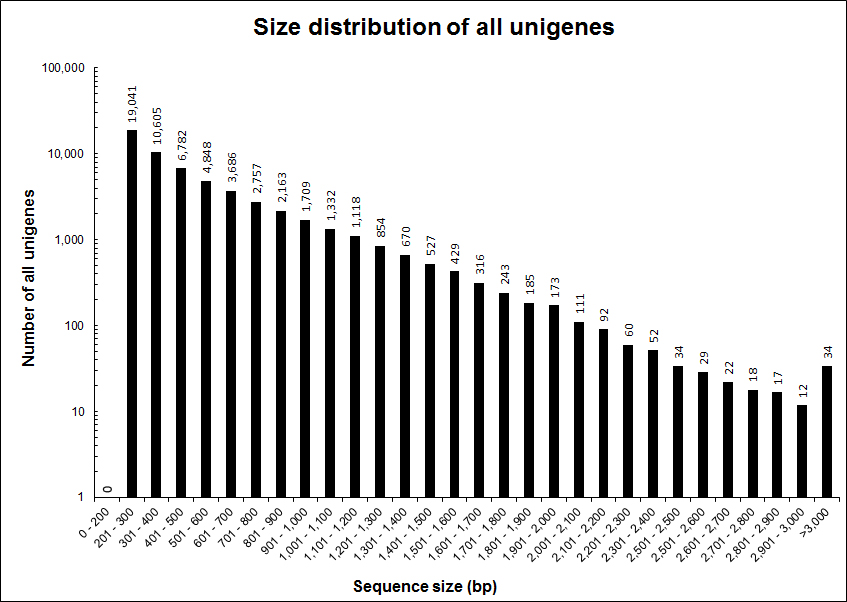


**Supplementary Figure S2. qRT-PCR analysis of anthocyanin biosynthetic genes during bilberry fruit development**. Relative expression of (a) CHS, (b) ANS, and (c) UFGT. Bars represent the relative expression levels of unigenes in each stage normalized with respect to the internal control *GAPDH*. Error bars represent standard error of four biological replicates. CHS, chalcone synthase; ANS, anthocyanidin synthase; UFGT, UDP-glucose flavonoid glucosyltransferase. S1 – Flower, S2 – Small unripe green fruit, S3 – Large unripe green fruit (G), S4 – Ripening purple fruit (R), S5 – Fully ripe blue fruit. G and R indicated two bilberry stages which are used for construction of transcriptome libraries. *Asterisks* indicate significant differences between early stage (S3) and ripening stages (S4, S5) at level p < 0.05^*^, p < 0.01^**^, p < 0.001^***^ using Student’s *t*-Test.


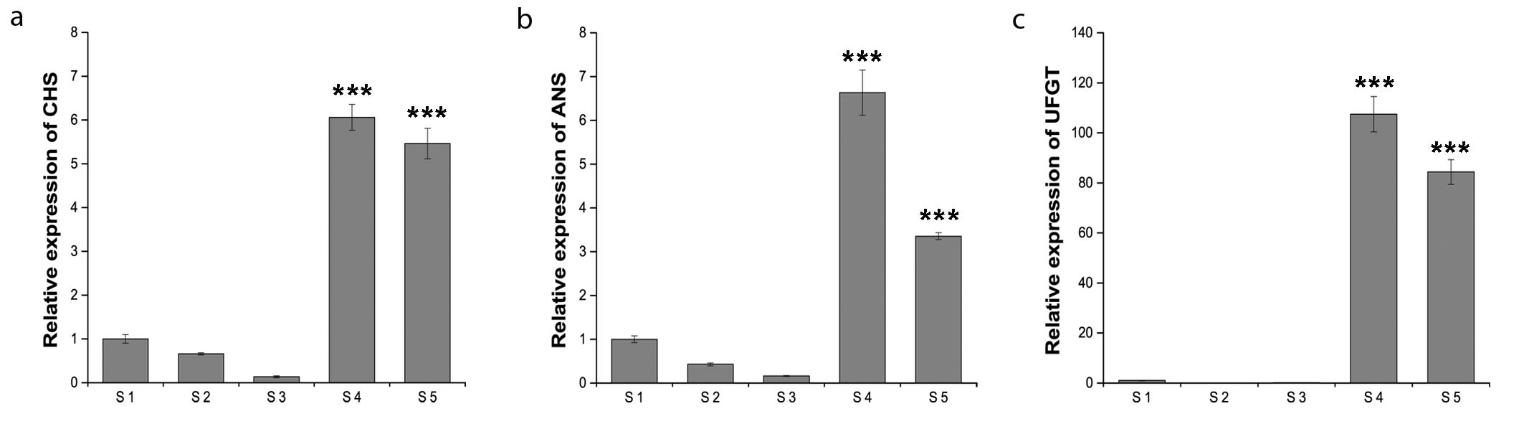


**References**

1. Dong, T. *et al*. A tomato MADS-box transcription factor, SlMADS1, acts as a negative regulator of fruit ripening. *Plant Physiol*. **163,** 1026-1036 (2013).
2. Tieman, D. M., Ciardi, J. A., Taylor, M. G. & Klee, H. J. Members of the tomato LeEIL (EIN3‐like) gene family are functionally redundant and regulate ethylene responses throughout plant development. *Plant J*. **26,** 47-58 (2001).
